# Taxonomic and mechanistic insights into gut microbiota bioaccumulation of entacapone using bioorthogonal drug labelling

**DOI:** 10.20517/mrr.2025.73

**Published:** 2025-11-20

**Authors:** Linda M. Guantai, Clementine E. Bavinton, Juwairiyah B. Shazzad, Sumeet Mahajan, Sam Thompson, Fatima C. Pereira

**Affiliations:** ^1^School of Biological Sciences, Faculty of Environmental and Life Sciences, University of Southampton, Southampton SO17 1BJ, United Kingdom.; ^2^School of Chemistry and Chemical Engineering, Faculty of Engineering and Physical Sciences, University of Southampton, Southampton, SO17 1BJ, United Kingdom.; ^3^Institute for Life Sciences, University of Southampton, Southampton SO17 1BJ, United Kingdom.

**Keywords:** Pharmaceuticals, bioorthogonal labelling, faecal microbiota, drug accumulation, siderophore transporter

## Abstract

**Aim:** The gut microbiota plays a key role in shaping individual responses to drugs, but current tools have limited potential to probe drug-microbe interactions within the complex, individualised gut environment. This study employed bioorthogonal labelling to track and identify gut microbial taxa and molecular mechanisms involved in the bioaccumulation of entacapone, a Parkinson’s disease drug.

**Methods:** We synthesised alkyne-tagged derivatives of entacapone and evaluated their suitability as molecular probes in *ex vivo* incubations with faecal communities or different *Escherichia coli* (*E. coli*) strains. Following incubation, tagged drugs were conjugated to a fluorescently labelled azide via click chemistry. Labelled cells were visualised, quantified, sorted via fluorescence-activated cell sorting (FACS), and identified via 16S ribosomal RNA (rRNA) gene amplicon sequencing.

**Results:** Entacapone alkyne derivatives retained the biological activity and effects of the original drug on the microbiota, significantly reducing microbial loads and shifting community composition across the three donors tested. Conjugation of alkyne-entacapone with a labelled azide revealed that between 80% to 96% of all microbial cells in a donor’s faecal sample accumulate entacapone. Nearly all taxa detected in incubations were recovered in labelled FACS fractions, confirming widespread uptake of the drug. Finally, we demonstrate that different *E. coli* strains exhibit varying levels of entacapone accumulation and identify a siderophore transporter that plays a role in this process.

**Conclusion:** Our findings reveal that entacapone is widely bioaccumulated by the gut microbiota across three donors and identify a key molecular mediator of this accumulation. This study expands the toolkit for investigating drug-microbiome interactions and holds significant potential to advance our understanding of drug-microbiome dynamics and therapeutic outcomes.

## INTRODUCTION

Drugs designed to target human cells constantly interact with microbes in the gastrointestinal tract^[1,2]^. These interactions can substantially shape the function of these microbial communities and may result in either beneficial or detrimental consequences for the host^[3-5]^. The Parkinson’s disease (PD) drug entacapone depletes bioavailable iron, a crucial micronutrient for most gut microbes^[2,6]^. This depletion suppresses commensals and favours iron-scavenging bacteria that harbour virulence and resistance genes, posing risks for PD patients^[2,7]^. In other instances, drug-induced changes to the gut microbiome have proven beneficial. For example, metformin, a drug used to treat type 2 diabetes, modulates the microbiome, resulting in increased short-chain fatty acid production - an effect that partially underlies metformin’s positive metabolic impact on the host^[8]^.

A key aspect of drug-microbe interactions is that microbes can also directly alter a drug’s pharmacokinetics, altering its bioavailability, efficacy, and toxicity^[9]^. This effect is primarily driven by microbial enzymatic modification of drugs^[4,10]^. However, microbes can also influence drug efficacy through bioaccumulation, a process involving the intracellular accumulation of drugs without chemical transformation, leading to reduced drug bioavailability and/or altered microbial community dynamics^[11,12]^. For instance, *Escherichia coli* (*E. coli*) IAI1 accumulates the antidepressant duloxetine, reducing its effect *in vivo*^[11]^. Duloxetine bioaccumulation also induces changes in metabolite secretion by *Streptococcus salivarius,* which in turn reshapes cross-feeding behaviours among gut bacteria^[11]^. In addition, bioaccumulation can alter community dynamics through cross-protection, wherein drug-sequestering microbes limit the exposure of neighbouring organisms^[12]^. Although observed across various gut microbial taxa, drug accumulation profiles can differ significantly even among closely related bacterial strains^[11]^. Most of the studies to date on drug bioaccumulation have focused on individual microbes or simplified communities^[11,12]^. Identifying the microbes within each individual's complex and highly personalised gut microbiome that are capable of drug accumulation could improve our ability to predict and manage drug-microbe interactions^[13]^.

Addressing drug bioaccumulation at a high resolution requires advanced, single-cell techniques that can link intracellular drug presence to microbial identity. For instance, drug bioaccumulation in microbial cells has been detected using stimulated Raman spectroscopy or microspectrofluorimetry^[2,14]^, which exploits the compound’s intrinsic photothermal or fluorescent signal. However, most drugs lack an intrinsic signal that can be exploited in this way. Fluorescent labelling is a viable alternative that enables high-throughput separation and identification of labelled cells via, e.g., fluorescence-activated cell sorting (FACS)^[15]^. However, conventional fluorophores are often bulky and can potentially alter drug permeability or function, limiting their effectiveness for *in situ* tracking^[16,17]^. A promising alternative is substrate analogue probing via bioorthogonal chemistry. In this approach, a small functional group, such as an alkyne moiety, is incorporated into a molecule to create a probe that reacts rapidly and selectively with an exogenous bioorthogonal handle^[18,19]^. This reaction enables the attachment of, e.g., fluorescent dyes, at a late stage in the experiment, allowing for the direct labelling of the drug in live microbial cells. Bioorthogonal labelling has already been applied in microbiome research. For example, it has been used to identify translationally active bacteria in human samples using bioorthogonal non-canonical amino acid tagging^[20,21]^ and to selectively image Gram-negative bacteria in the mouse gut via azide-modified lipopolysaccharide precursors^[22]^. However, the application of bioorthogonal chemistry to visualise drug bioaccumulation within microbiomes remains to be explored.

Here, we tested and applied bioorthogonal labelling combined with click chemistry to investigate entacapone bioaccumulation by microbes at a single-cell level. Entacapone was selected as the model drug because of its significant and well-characterised effects on the gut microbiome and the understudied nature of its bioaccumulation^[2]^. Two structural analogues of entacapone containing alkyne groups were synthesised *de novo*. Using click chemistry and FACS, microbial cells that accumulated the drug were fluorescently labelled, imaged, sorted, and DNA sequenced to identify entacapone-bioaccumulating taxa. The use of labelled derivatives also enabled the identification of a key molecular determinant of entacapone bioaccumulation. Our approach enables versatile *in situ* investigation of drug-microbe interactions, supporting the development of microbiome-informed strategies to improve drug efficacy in a personalised manner.

## METHODS

### Synthesis of alkyne-tagged entacapone derivatives

The synthesis of entacapone (entacapone^S^) and mono- and di-alkynyl derivatives EB-11-24 and EB-11-38, respectively, was based on a modification of a previously reported procedure^[23]^. Condensation of 3,4-dihydroxy-5-nitrobenzaldehyde and diethylcyanoacetamide, which are both commercially available, gave entacapone^S^. Substituting diethylcyanoacetamide with 2-cyano-*N*-(prop-2-yn-1-yl) acetamide or 2-cyano-N, N-di(prop-2-yn-1-yl) acetamide gave EB-11-24 and EB-11-38 respectively. Descriptions of synthetic procedures, compound characterisation, and spectral data are available in Supplementary Materials.

### Sample collection

Fresh faecal samples were collected from three healthy adults (two females and one male). Study participants were aged between 25 and 42 years and had not received antibiotics in the last three months. The study protocol was approved by the University of Southampton Ethics Committee (ERGO II 78743). The participants were provided with a faecal collection kit including a sterile faecal collection container with a collection spoon (Sarstedt, Numbrecht, DE) and a sterile faeces catcher (FecesCatcher, Tag Hemi, Zeijin, NL), placed inside a BD GasPak**^TM^** EZ Gas Generating system pouch. The participants did not receive any compensation for participating in this study. They signed an informed consent, and participant metadata is 100% pseudo-anonymised.

### *Ex vivo* incubations with entacapone

Samples were transferred into an anaerobic chamber (5% H_2_, 10% CO_2_, 85% N_2_, Coy Laboratory Products, USA) within 90 min after defecation, and subsequently resuspended with 10 mL supplemented M9 mineral medium^[24]^ (sM9) composed of 0.5 mg. mL^-1^ D-glucose, 0.1% L-cysteine, 0.5% (v/v) of vitamin solution, and 0.1% (v/v) mineral solution (both prepared according to DSMZ, Medium 461). The samples were vortexed for 30 s and then left to settle for 10 min to allow the larger particles to deposit at the bottom of the vial. Then, 1 mL of the pre-settled mixture was transferred to a new 50 mL Falcon tube and diluted 10 times in sM9. Native and modified entacapone molecules were pre-dissolved in dimethyl sulfoxide (DMSO, Merck) and supplemented to the faecal slurries to reach a final concentration of 1,965 μM of drug and 1% DMSO (v/v). This is the estimated entacapone concentration in the colon based on a typical oral dose of 655.1 μmol^[1]^(equivalent to 200 mg), taking into account the molecular weight of entacapone (305.286 g.mol^-1^). A no-drug control, where DMSO alone was supplemented to faecal samples for the final 1% (v/v), was also included. Incubations were established in replicates. Sample aliquots were collected at 0, 6, and 24 h of incubation under anaerobic conditions at 37 °C. A 40 µL aliquot was mixed with phosphate-buffered saline (PBS) containing glycerol for a final 20% glycerol in the vial and immediately stored at -80 °C. A second aliquot (40 µL) was pelleted by centrifugation for 5 min at 12,000 *g* and immediately stored at -20 °C for subsequent nucleic acid extraction. Finally, a 400 μL sample aliquot was collected at 4 and 6 h, pelleted by centrifugation at 10,000 × g, washed with 1× PBS, fixed in 50% ethanol, and stored at -20 °C until further use.

### Cell counts using flow cytometry

Microbial cell counts in faecal samples preserved in 20% PBS glycerol were assessed using flow cytometry [Supplementary Table 1]. Samples were initially diluted 1:100 in filtered 1× PBS, and nucleic acids were stained by adding 0.5 μM SYTO**^TM^** Deep Red nucleic acid stain (Thermo Fisher Scientific, UK), followed by incubation in the dark for 15 min. Subsequently, the samples underwent analysis with a Guava easyCyte**^TM^** cytometer (Merck) and were processed with Guava® InCyte software. Cell counts were determined based on the presence of SYTO**^TM^** Deep Red signal using a 642 nm laser. Gating on SYTO**^TM^** Deep Red dye was established based on an unstained control sample and a stained positive control, (gating strategy is exemplified in Supplementary Figure 1A).

### DNA extraction from faecal samples

Nucleic acids in pellets from samples incubated with entacapone and derivatives were extracted using the InnuPREP DNA/RNA Mini Kit (Analytik Jena, Germany). First, 600 μL of Lysis Solution RL was added to the pelleted faecal bacteria, and the pellet was resuspended. Then, 600 μL of the sample was transferred into the Lysis Buffer Solution RL in the screw-cap Invitrogen Bead tubes (Thermo Scientific). Homogenisation was performed on a QIAGEN tissue lyser LT (QIAGEN, Germany) at 40 Hz for 3 × 30 s, with cooling on ice between each cycle. The tubes were centrifuged at room temperature for 10 min at 10,000 *g*. Next, the supernatant was transferred into a Spin Filter D (InnuPREP DNA/RNA Mini Kit, Analytik Jena, Germany) and placed into a receiver tube, as extraction was carried out according to the manufacturer’s instructions. DNA was eluted in 30µL of nuclease-free water. Negative controls included DNA extraction from filtered 1× PBS, performed in parallel with the samples.

### 16S rRNA gene amplicon sequencing

Polymerase chain reaction (PCR) amplification, library preparation, and amplicon sequencing were performed by Novogene. The primers 515F (5′-GTGCCAGCMGCCGCGGTAA-3′) and 806R (5′-GGACTACHVGGGTWTCTAAT-3′)^[25]^ were used for the study. All PCR reactions were conducted with Phusion® High-Fidelity PCR Master Mix (New England Biolabs), 2 μM of forward and reverse primers, and approximately 10 ng of template DNA. Thermal cycling included an initial denaturation at 98 °C for 1 min, followed by 30 cycles of denaturation at 98 °C for 10 s, annealing at 50 °C for 30 s, and elongation at 72 °C for 30 s, with a final extension at 72 °C for 5 min. Sequencing libraries were prepared using the TruSeq® DNA PCR-Free Sample Preparation Kit (Illumina, USA), following the manufacturer's recommendations. The library quality was assessed on the Qubit 2.0 Fluorometer (Thermo Scientific) and Agilent Bioanalyzer 2100 system. The library was sequenced on an Illumina NovaSeq platform, 2 × 250 bp mode. Paired-end reads were demultiplexed using Python (v3.6.13) and filtered with cutadapt (v3.3)^[26]^. Denoising into amplicon sequencing variants (ASVs) was performed with DADA2^[27]^ using a previously described standard protocol^[28]^. FASTQ reads 1 and 2 were trimmed at 220 nt and 150 nt, with allowed expected errors of 2. Taxonomy was assigned to 16S ribosomal RNA (rRNA) gene/transcript sequences based on SILVA taxonomy (release 138) using Mothur^[29]^.

All samples were processed using the vegan package (v2.6.6.1)^[30]^ and phyloseq (v1.46.0)^[31]^ within the R software environment (https://www.r-project.org/, R 4.3.1). ASVs with fewer than 30 reads were pruned. Subsequently, ASVs belonging to taxa not typically found in the human gut microbiome and with low read counts per sample were removed, including the genera *Ralstonia* (4 ASVs), *Mesorhizobium* (2 ASVs), *Bradyrhizobium* (4 ASVs) and *Chloroplast_insertae_sedis* (14 ASVs,), and from the phyla *Patescibacteria* (10 ASVs), *Planctomycetota* (4 ASVs), and *Deinococcota* (1 ASV) [Supplementary Table 2]. A total of 1605 ASVs were retained for analysis. The average read count per sample was 54771 ± 15354, and sample coverage was above 99% for all samples [Supplementary Table 2]. For quantitative microbiome analysis, relative abundances were adjusted for 16S rRNA gene copy number using rrnDB (version 5.9), applying the rrnDB-5.9 PantaxaStats dataset for correction^[32]^. Relative abundances of ASV, after copy number correction [Supplementary Table 3 and Supplementary Figure 2], were multiplied by microbial cell counts from flow cytometry to calculate absolute abundances [Supplementary Table 4]. DESeq2 (v1.46.0)^[33]^, implemented in phyloseq, was used to identify taxa significantly affected by Entacapone^s^, EB-11-24, and EB-11-38. Only 488 ASVs with more than 1.0 × 10^6^ reads (quantitative microbial profile) were considered for DESeq2 analysis [Supplementary Table 5].

### Azide-alkyne click reaction

Cu(I)-catalysed click labelling of chemically fixed microbial cells was performed in solution according to^[34]^. The azide-alkyne click reaction was achieved via a Cu(I)-catalysed reaction where the terminal alkyne group(s) of EB-11-24 and EB-11-38 are coupled to an Alexa Fluor**^TM^** 488 azide (AF488-azide) fluorescent dye (Jena Bioscience, Germany), yielding a triazole^[35]^. In brief, ethanol-fixed samples from 4- and 6-h time intervals were washed with 96% ethanol and then resuspended in 221 μL of 1× PBS. 12.5 μL of a freshly prepared 5 mM sodium ascorbate solution and 5 mM aminoguanidine hydrochloride solution were added to the sample and mixed by vortexing. A dye-mix solution was prepared by mixing 100 µM CuSO_4_, 500 µM THPTA, and 5µM azide dye (Jena Bioscience, Germany). The mixture was incubated for 3 min at room temperature in the dark and then added to the sample. After inversion to mix the dye, the tubes were incubated in the dark at room temperature for 30 min. The cells were then washed twice in 500 μL 1× PBS, and the pellet was finally resuspended in 50 µL PBS.

### FACS

Freshly clicked samples were diluted 1:100 with filtered sterile 1× PBS. Subsequently, the cells underwent staining with 0.5 μΜ SYTO**^TM^** Deep Red fluorescent dye for 15 min in the dark. Samples were subsequently sorted into two separate fractions using a BD FACSAria IIu cell sorter (BD Biosciences). The two cell fractions consisted of a SYTO**^TM^** Deep Red positive cell fraction (SYTOPOS; this includes all SYTO positive cells irrespective of the Alexa Fluor**^TM^** 488 signal), and a SYTO**^TM^** Deep Red positive and Alexa Fluor**^TM^** 488-labelled cells (AZIDEPOS; including only SYTO and Alexa Fluor**^TM^** 488 positive cells). Briefly, background signals from the instrument and the buffer solution (PBS) were identified using the operational parameters forward scatter (FSC) and side scatter (SSC). Microbial cells were then displayed with the same settings in a scatterplot using the FSC and SSC, and pre-gated based on the presence of SYTO**^TM^** Deep Red signals (for gating strategy, please see Supplementary Figure 3), detected using the red (647 nm) optical laser. To determine the gate for AZIDEPOS cells, a blue laser was used (488 nm), in addition to the fluorescence detected with a 647 nm laser. Cells (between 12,500 and 25,000 cells per sort) were sorted into 1.5 mL Eppendorf tubes filled with 100 μL filtered sterile PBS. The samples were stored at -80 °C freezer for nucleic acid extraction, amplification, and sequencing (see Supplementary Materials).

### Statistical analyses

All statistical analyses were performed using R version 4.3.2. Normality was assessed with the Shapiro-Wilk test (*P* > 0.05 indicated normal distribution)^[36]^. For normally distributed data with homogeneous variances (Levene’s test), one-way analysis of variance (ANOVA) followed by Tukey’s Honest Significant Difference (HSD) test was used for pairwise comparisons. When assumptions were violated, the Kruskal-Wallis test followed by Dunn’s test with Bonferroni correction was applied^[37,38]^. Microbial community composition was analysed using permutational multivariate analysis of variance (PERMANOVA) on Bray-Curtis dissimilarities (vegan package) to assess the effects of drug treatment and donor, with significance determined by permutation.

Additional methods describing details of fluorescence microscopy, fluorescence signal measurements, and determination of AF488-positive cells by flow cytometry can be found in Supplementary Materials.

## RESULTS

### Chemical synthesis of alkyne-tagged entacapone derivatives

**Figure 1:**
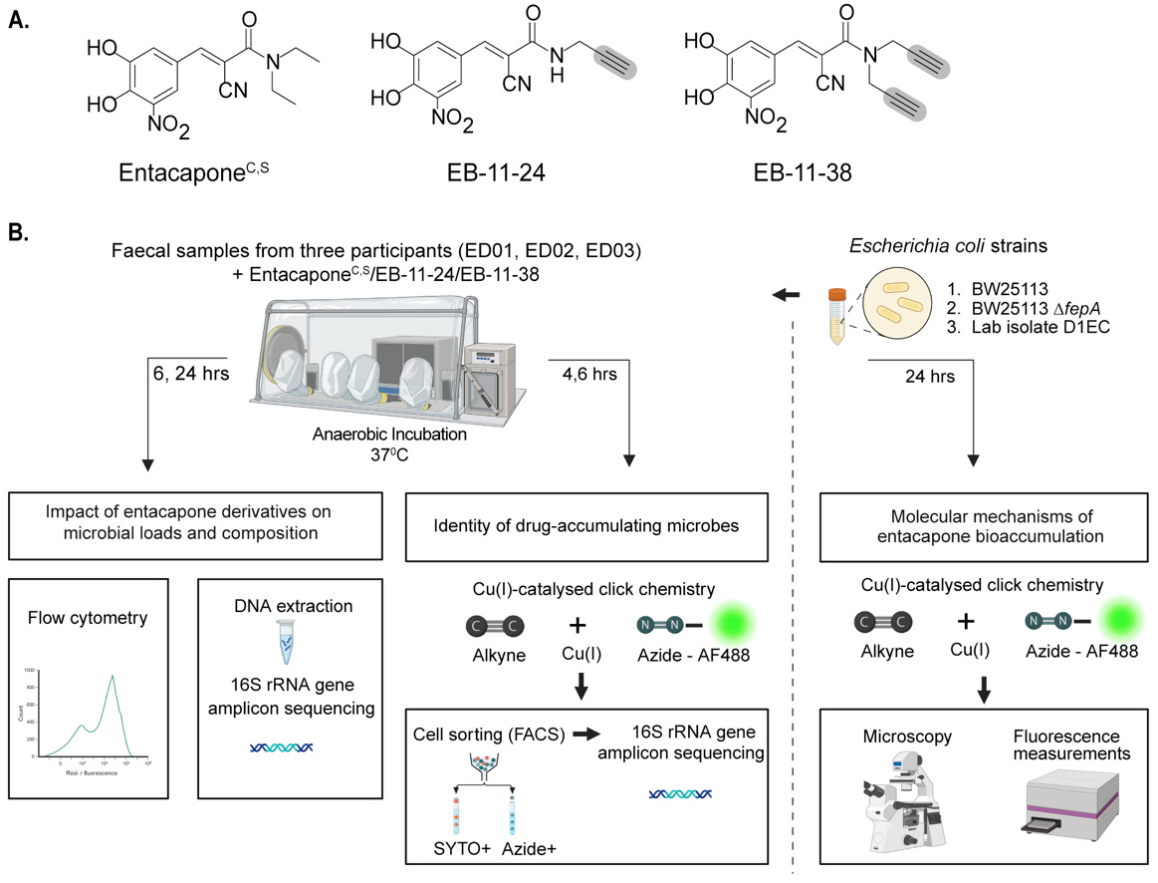
Bioorthogonal labelling combined with click chemistry to investigate entacapone bioaccumulation by microbes at the single-cell level. (A) Chemical structures of native entacapone (entacapone^C^), and newly synthesised entacapone (entacapone^S^) and alkynyl entacapone derivatives EB-11-24 (1 alkyne) and EB-11-38 (2 alkynes). Structural modifications to the native entacapone molecule include one or two terminal alkyne groups, highlighted in grey; (B) *Ex vivo* anaerobic incubation and processing of entacapone derivatives. Faecal samples from three healthy participants (ED01, ED02, ED03) were incubated under anaerobic conditions and supplemented with entacapone or its derivatives. Microbiome composition and abundance were assessed through flow cytometry and 16S rRNA gene sequencing at 6- and 24-h timepoints. Drug-responsive microbes were identified using click-chemistry and fluorescence-activated cell sorting (FACS) at 4- and 6-h timepoints, followed by 16S rRNA gene sequencing. *E. coli* strains were incubated with entacapone derivatives under anaerobic conditions and subjected to click-chemistry and fluorescence measurements to determine molecular cues of entacapone accumulation. This figure was created in Biorender (https://BioRender.com/xhx4f6v). rRNA: Ribosomal RNA; FACS: fluorescence-activated cell sorting.

Entacapone bioaccumulates in microbial cells and can be detected by stimulated Raman spectroscopy, through its intrinsic photothermal signal^[2]^. However, this approach alone cannot comprehensively identify all microbial taxa interacting with the drug. To link single-cell drug bioaccumulation to phylogenetic identity in complex communities, we tested and applied a bioorthogonal labelling approach combined with click chemistry. Two structural analogues of entacapone containing alkyne groups were synthesised *de novo*, alongside the native entacapone molecule (entacapones^S^, Figure 1A, Supplementary Materials). Commercial entacapone (entacapone^C^, Figure 1A) has previously been shown to impact microbial growth and reduce the abundance of key microbes by chelating iron through its catechol group^[2]^. Therefore, alkyne groups were incorporated into the entacapone molecule away from the catechol group. A first variant contains a single terminal alkyne group replacing a methyl group (EB-11-24; Figure 1A). To maximise the chances of achieving sufficient labelling, a second variant was synthesised in which both terminal methyl groups were replaced with terminal alkynes (EB-11-38; Figure 1A).

### Alkyne-tagged entacapone derivatives retain the biological activity of native entacapone

**Figure 2:**
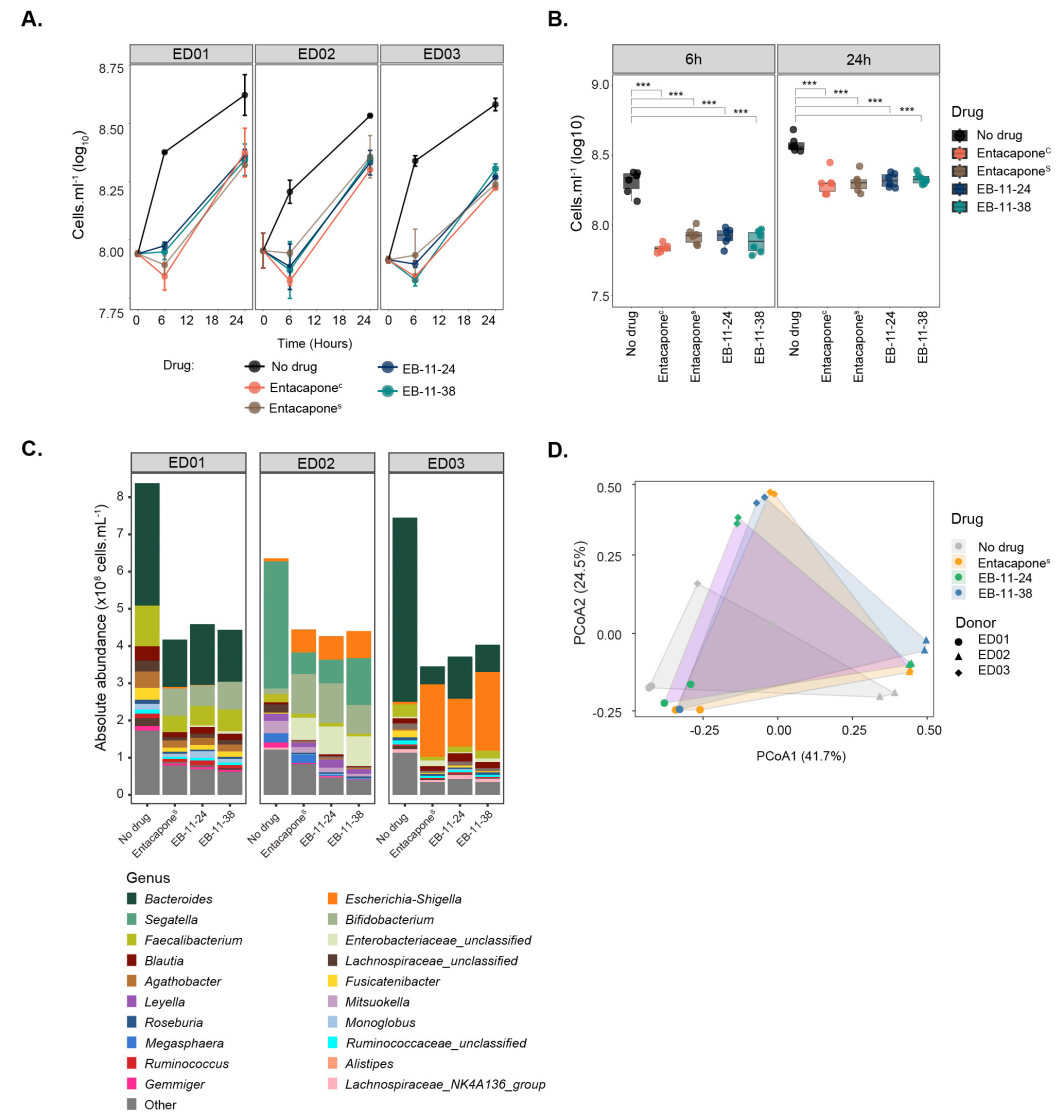
Entacapone alkyne-derivatives impact microbiota composition and abundance in a manner similar to the native drug. (A) Microbial cell loads in faecal incubation vials amended with entacapone^C^, entacapone^S^, EB-11-24 and EB-11-38, and a no-drug control (DMSO). Samples originated from three different donors (ED01, ED02, or ED03). Microbial cell loads were measured at 0, 6, and 24 h by flow cytometry; (B) Box plots illustrate the significant impact of entacapone derivatives and commercial entacapone on microbial growth, as shown in A. Error bars indicate the standard deviation from duplicate samples. Statistical significance between drug-treated samples and the DMSO no-drug control was assessed using one-way ANOVA with Tukey’s HSD test, (*P* < 0.001***), as presented in Supplementary Table 1; (C) Absolute abundances of genera assessed using 16S rRNA gene amplicon sequencing in no-drug controls and samples treated with entacapone^S^, and EB-11-24, EB-11-38, separated per donor. For absolute abundances, genera with counts below 3.0 × 10^6^ cells. mL^-1^ were categorised as “Other”; (D) Principal Coordinate Analysis (PCoA) of gut microbiota community composition for drug-treated samples and controls across three donors, based on a Bray-Curtis dissimilarity matrix. HSD: Honest significant difference; ANOVA: analysis of variance.

While biorthogonal labelling is generally considered inert, *i.e*., to retain the biological activity of the native molecule, microbial transformation of the drug and/or structural interference from the label can occur^[10]^. These factors may alter the labelled drug’s structure or activity, potentially limiting its suitability for studying drug-microbe interactions. To assess whether the alkyne drug variants retain biological activity, batch incubations with drugs were established using faecal samples originating from different donors [Figure 1B]. Microbial cell counts revealed significantly lower microbial loads for samples treated with either entacapone^C^, entacapone^S^, EB-11-24, or EB-11-38 compared to the no-drug control after 6 and 24 h of incubation (Figure 2A and B, *P* < 0.001, one-way ANOVA with Tukey’s HSD test). In line with previous findings^[2]^, a slight recovery in the growth of all entacapone-treated samples was observed after 24 h of incubation across all donors, and this was mirrored by the two alkyne derivatives [Figure 2A and B]. No statistically significant differences were observed between samples treated with entacapone^C^ and entacapone^S^ at either time point, indicating that the synthetic compound matches the commercial standard in quality and biological effect [Figure 2B and Supplementary Table 1]. Importantly, no statistically significant differences in microbial loads were found when comparing entacapone^S^, EB-11-24, and EB-11-38 samples at any of the analysed time points [Figure 2B and Supplementary Table 1]. This demonstrates that alkyne derivatives have a similar effect on microbial growth to that of native entacapone.

Previous work reported that entacapone can alter the composition of the microbiota, decreasing the abundance of protective species, including *Bacteroides*, *Clostridium,* or *Faecalibacterium*, while increasing the abundance of *Escherichia/Shigella*, *Bifidobacterium,* or *Ruminococcus*^[2,39]^. To investigate in detail the ability of alkyne-derivatives to mimic the biological effects of the native entacapone on microbial communities from different donors, drug-incubated faecal samples were analysed using 16S rRNA gene amplicon sequencing [Supplementary Table 2]. Relative abundance profiles were integrated with microbial cell counts to calculate absolute abundances^[40]^ of each taxon within the community for the three different sample donors [Supplementary Tables 3 and 4, Figure 2C]. While *Segatella* (formerly classified as *Prevotella*) was the most abundant genus detected in control (no drug) samples from donor ED02, controls from donors ED01 and ED03 were dominated by *Bacteroides* [Figure 2C]. PERMANOVA analysis revealed that both drug treatment and donor significantly influenced the microbial community composition (PERMANOVA by drug: R^2^ = 0.122, *P* = 0.004; PERMANOVA by donor: R^2^ = 0.628, *P* = 0.001). Significant differences in microbial community composition were observed between the no-drug control and all treatment groups. Specifically, entacapone^S^ (*P* = 0.015, R^2^ = 0.207), EB-11-24 (*P* = 0.030, R^2^ = 0.186), and EB-11-38 (*P* = 0.048, R^2^ = 0.176) all differed significantly from the control. However, no significant differences were observed between the drug treatment groups themselves (*P* > 0.05), suggesting similar effects of either alkyne-drug derivative on microbiota community composition compared to native entacapone [Figure 2D].

**Figure 3:**
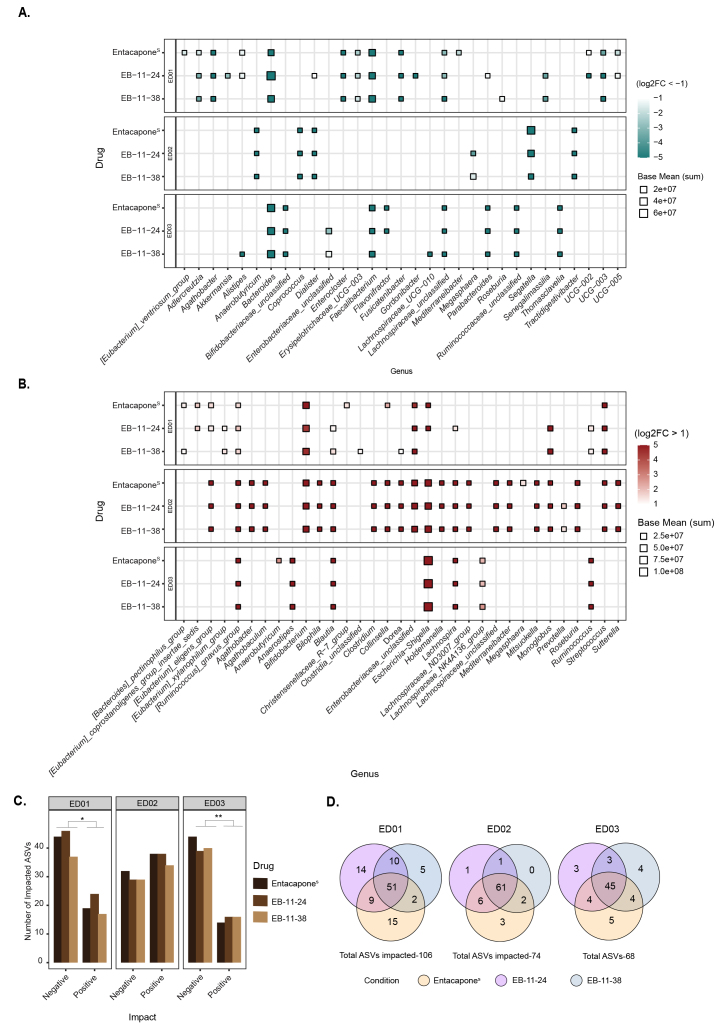
Faecal microbial taxa significantly affected by entacapone and its derivatives. (A) Heatmap depicting genera differentially abundant across treatment conditions (entacapone^S^, EB-11-24, EB-11-38) for each donor, faceted by rows. The x-axis represents microbial genera that were significantly decreased under each treatment compared to no-drug (log_2_FC < -1, *P_adjusted_* < 0.05, DESeq2 analysis), while the y-axis represents the respective treatment; (B) Genera that were significantly increased in abundance under each treatment condition relative to the no-drug control (log_2_FC > 1, *P_adjusted_* < 0.05, DESeq2 analysis), across all donors. In A and B, point size indicates the average abundance (base mean), and colour represents log_2_FC with values capped at < -5 and > 5 to enhance visual clarity. “i.s.”: *incertae sedis*; (C) Bar plot depicting the number of ASVs either positively or negatively affected by each treatment across individual donors. The x-axis indicates the direction of impact, while the y-axis represents the number of significantly affected ASVs. Bars are coloured by drug treatment; (D) Venn diagrams illustrating the number of affected ASVs unique to entacapone^S^ (yellow), EB-11-24 (purple), EB-11-38 (blue), and shared between the conditions. Total ASV counts per donor are indicated. ASV: Amplicon sequence variant.

At the genus level, drug treatment of ED01 and ED03 samples was marked by a significant reduction in *Bacteroides*, *Faecalibacterium, and Lachnospiraceae* unclassified (DESeq2 analysis, Figure 3A). Additional genera such as *Agathobacter, Fusicatenibacter*, and *Enterocloster* were significantly decreased in ED01, while in ED03 there was an additional decrease in *Parabacteroides*, *Ruminococcaceae* unclassified, *Bifidobacteriaceae unclassified* and *Thomasclaveliae*. Increases in *Escherichia/Shigella, Blautia, and Ruminococcus gnavus* were observed across the three donors [Figure 3B]. In ED01, these increases are accompanied by an increase in *Enterobacteriaceae*, *Bifidobacterium,* and *Streptococcus.* An analysis at higher taxonomic resolution (ASV-level) also revealed that donor-specific variations were observed, with 106, 74, and 68 ASVs significantly (*P* < 0.05) affected in ED01, ED02, and ED03, respectively [Figure 3C and D]. For donors ED01 and ED03, the number of ASVs whose abundance is negatively impacted by entacapone surpasses the number of ASVs whose abundance is positively impacted [Figure 3C and Supplementary Figure 4]. In contrast, donor ED02 exhibited the opposite pattern [Figure 3C and Supplementary Figure 4]. Indeed, in donor ED02 several genera, including Agathobacter and the Eubacterium eligens group, show an increase in abundance compared to the control [Figure 3B]. The number of ASVs affected, both positively and negatively, by either drug derivative or entacapone^S^ is largely consistent across all donors, though results for ED01 show greater variability [Figure 3C and D]. Indeed, while ED01 shows agreement among the most abundant and significantly impacted ASVs, some variability appears to be driven by ASVs with smaller Log_2_FC changes [Supplementary Figure 4 and Supplementary Table 5]. Overall, our results indicate that the structural similarity of the alkyne derivatives is sufficient to preserve their functional properties, confirming that they retain substantial biological activity.

### Alkyne-tagged entacapone enables tracking of drug incorporation at single-cell resolution

**Figure 4:**
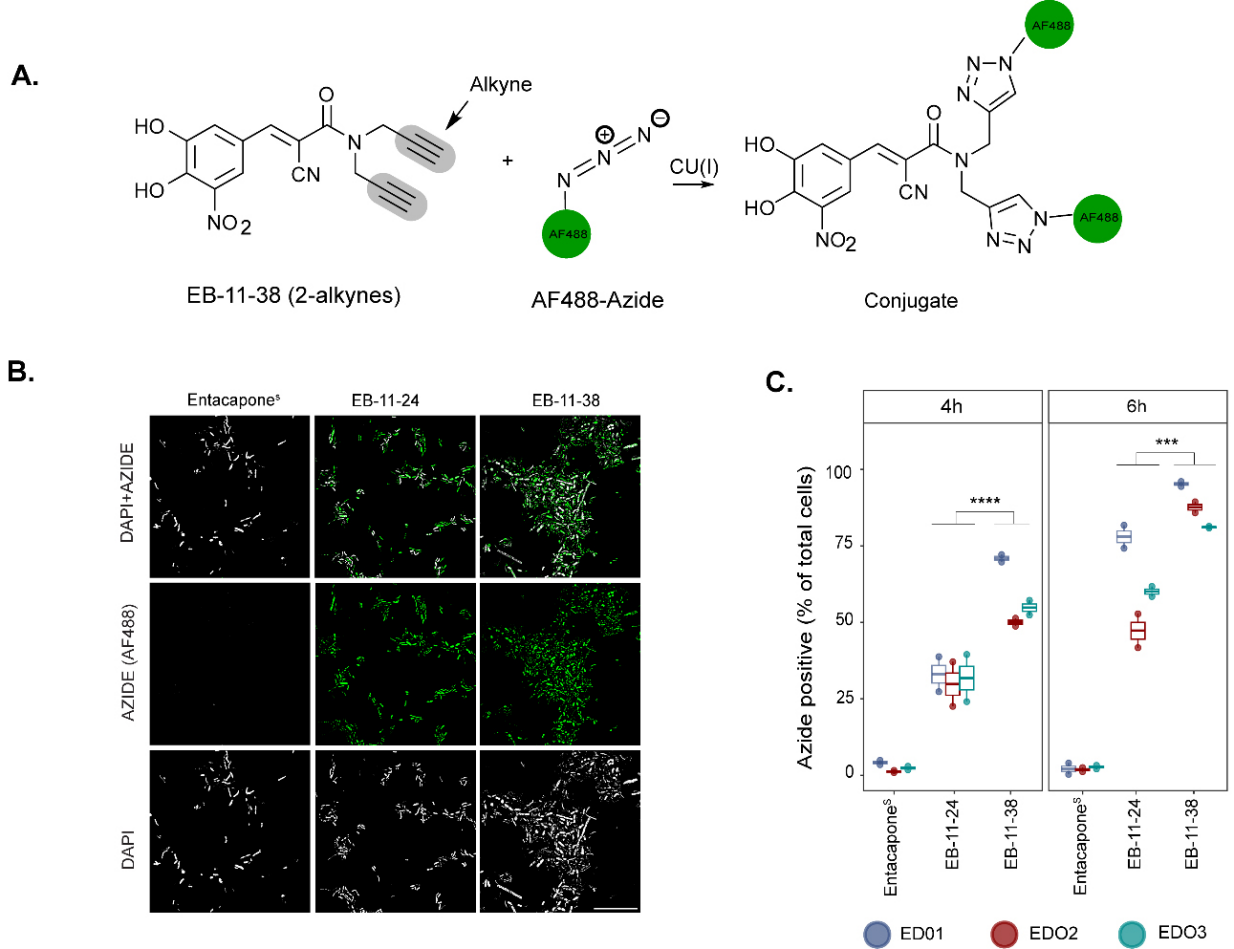
Click chemistry of alkyne-tagged entacapone enables tracking of drug incorporation at single-cell resolution. (A) The schematic representation shows copper-catalysed conjugation of entacapone alkyne derivatives with an AF488-labelled azide; (B) Microscope images showing AF488 signal. DAPI and AF488 azide fluorescence signals are shown in grey and green, respectively. Scale bar: 30 µm; (C) Box plot depicting the percentage of AF488-labelled microbial cells, indicating accumulation of the derivatives, colour coded by donor, at 2 incubation time points (4 h and 6 h). Data points represent replicates for each of the three donors per drug condition (*n* = 6 for each drug condition). Statistical significance between drug conditions was assessed using a Tukey’s post-hoc test following ANOVA. The comparison between EB-11-24 and EB-11-38 resulted in a *P*-value of < 0.05 at both timepoints. All comparisons were made with a 95% confidence interval. AF: Alexa Fluor; ANOVA: analysis of variance.

To follow drug accumulation by microbial cells, faecal samples incubated with alkyne-tagged derivatives were subjected to copper-catalysed click-chemistry^[34,41]^ conjugating the drug to a fluorescently labelled azide (AF488-azide, Figure 4A). We focused our analyses on the initial incubation time points (4 and 6 h) to capture the microbial community's response to the drug before any significant changes in community composition occur. Fluorescence microscopy and flow cytometry analyses confirmed the presence of AF488 fluorescence signal originating from both EB-11-24 and EB-11-38 in microbial cells, while clicked entacapone^S^-incubated cells showed no detectable signal [Figure 4B and C]. Additionally, signal was observed only in a very small fraction of cells that had been fixed before incubation with either drug derivative (that is, dead cells) [Supplementary Table 6]. A significantly higher percentage of azide-positive cells was observed following incubation with EB-11-38 compared to EB-11-24 or entacapone^S^ at either time point (*P* = 3.49 × 10^-5^ and *P* = 4.97 × 10^-4^ for 4 and 6 h of incubation, respectively; ANOVA with post hoc Tukey’s HSD). This suggests that the presence of two alkyne moieties enhanced signal detection compared to one alkyne [Figure 4C]. As expected, for each sample, a higher percentage of AF488 azide-positive cells was observed after 6 h of incubation compared to 4 h, indicating that drug bioaccumulation builds over time [Figure 4C]. ED01 incubated with EB-11-38 exhibited the highest percentage of AF488azide-positive cells (94%), followed by ED02 (87%), while ED03 displayed the lowest percentage (81%) at the 6-h time point [Figure 4C and Supplementary Table 6]. These findings confirm that alkyne labelling via click chemistry enables the detection and visualisation of drug accumulation within the gut microbiota. Although entacapone broadly accumulates in faecal microbial cells, the proportion of accumulating cells varies between donors, likely reflecting differences in baseline community composition due to interindividual variability commonly observed in the population.

**Figure 5:**
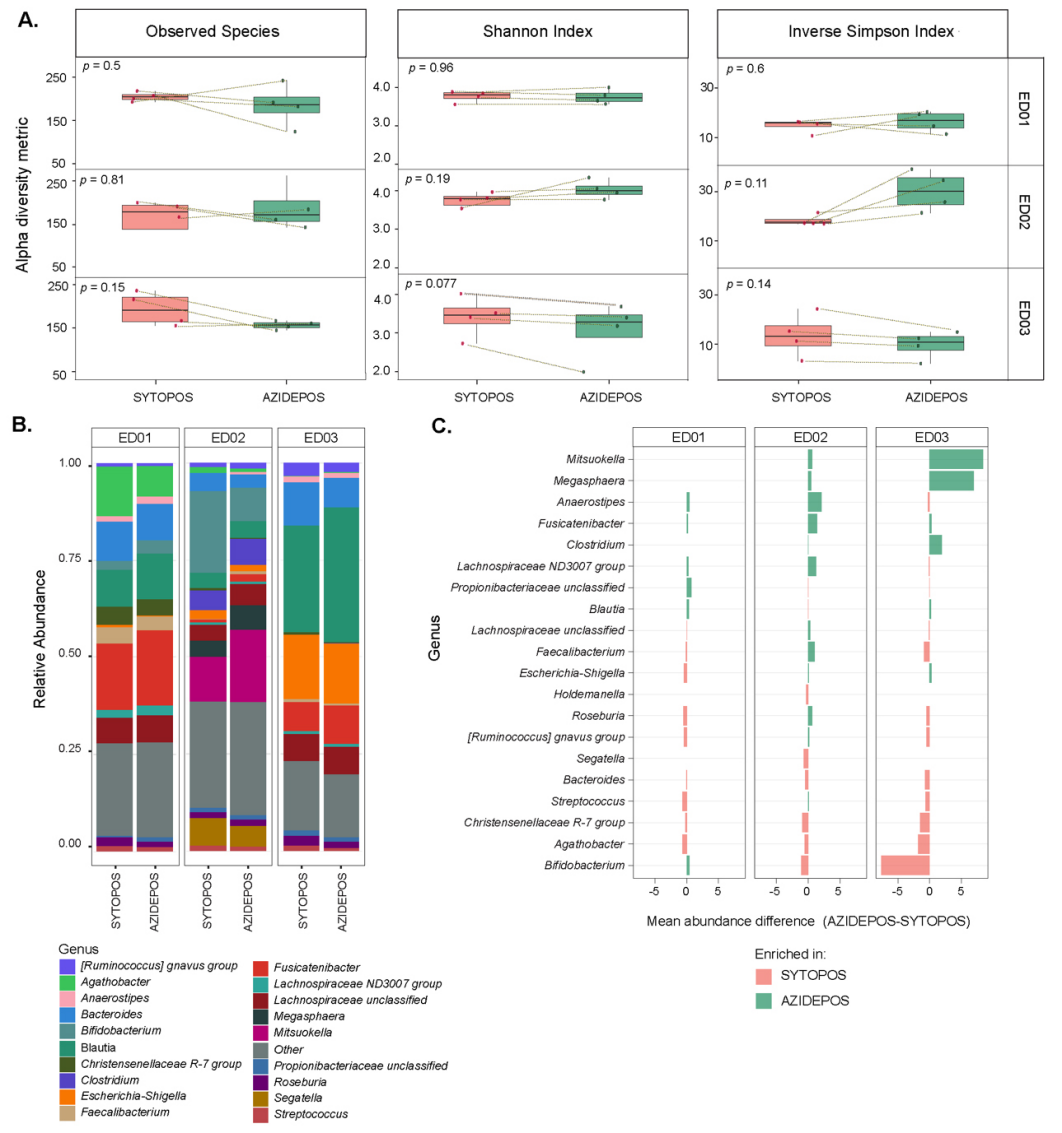
Microbial community diversity and composition of drug-associated or total microbial cell fractions sorted by FACS. (A) Alpha diversity metrics across sorted cell populations. For each metric, samples from the same donor were compared between SYTOPOS (total microbial cells) and AZIDEPOS (EB-11-38-associated cell populations) fractions (paired cell sorts from the same sample). Statistical significance was evaluated using paired *t*-tests, and *P*-values are shown on each plot. Panels are facetted by donor; (B) Relative abundance of bacterial genera across samples sorted by SYTOPOS and AZIDEPOS populations at 4- and 6-h timepoints. Bar plots show the relative abundance of the top 20 bacterial genera (y-axis) within each sorted sample (x-axis), grouped by donor and treatment condition. Genera not among the top 20 are grouped under “Other”; (C) Bar plot depicting the difference in mean relative abundance of microbial genera in AZIDEPOS and SYTOPOS populations across three donors at 4- and 6-h timepoints. Each bar represents a genus, with colours indicating whether the genus is more abundant in drug-associated cells (green) or in the total microbial community (light red). The x-axis lists microbial genera, while the y-axis represents the difference in abundance (AZIDEPOS minus SYTOPOS). *T*>-test analysis revealed no significant differences in genera abundance between sorted fractions (all comparisons: *P* < 0.05). FACS: Fluorescence-activated cell sorting.

To determine the phylogenetic identity of cells that accumulate entacapone, samples incubated with EB-11-38 and displaying AF488-azide signal were sorted using FACS, and sorted fractions were subjected to 16S rRNA gene amplicon sequencing. [Supplementary Figure 3]. For each sample, paired SYTO-positive fractions, *i.e*., all cells stained with the nuclei dye SYTO**^TM^** Deep Red, independently of the AF488 signal, were also sorted and sequenced. Alpha diversity analyses [Supplementary Table 7] revealed that a similar number of species can be recovered from AF488-positive versus SYTO-positive fractions for all donors [Figure 5A], suggesting that entacapone interacts broadly with the gut microbiota rather than accumulating in a selective subset of taxa. Nevertheless, taxonomic analysis revealed several genera with differing relative abundances [Supplementary Table 8] between AF488-positive and SYTO-positive fractions [Figure 5B and C]. *Fusicatenibacter* was consistently enriched in the AF488-positive group across all three donors. *Anaerostipes* and *Lachnospiraceae* ND3007 group were enriched in AF488-positive of ED01 and ED02, while *Mitsoukella, Megasphaera,* and *Clostridium* were enriched in AF488-positive groups of ED02 and ED03 [Figure 5B and C, Supplementary Table 9]. On the contrary, taxa such as *Bifidobacterium*, *Agathobacter,* and *Christensenellaceae* R-7 group were more abundant in SYTO-positive fractions [Figure 5C]. The lack of significant differences supports the view that entacapone interacts broadly with virtually all microbial taxa of different donors. The different levels of cells displaying a signal may indicate phenotypic variants across various taxa.

### Identification of molecular determinants of entacapone accumulation

**Figure 6:**
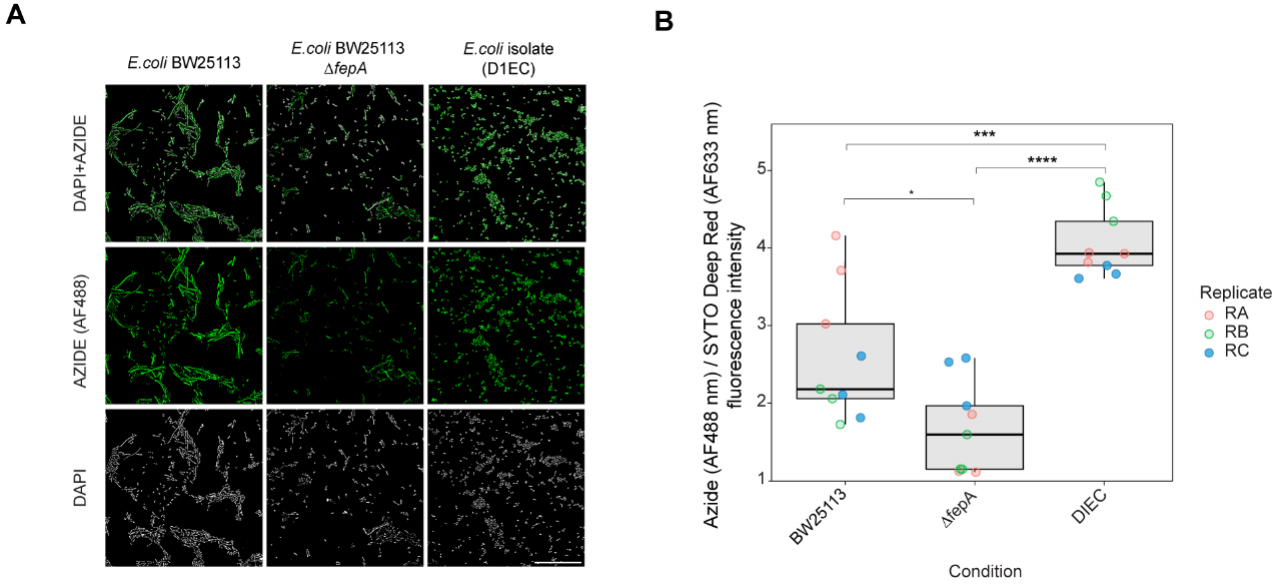
Identification of molecular determinants of entacapone accumulation in *E. coli*. (A) Microscopy images showing the interaction of EB-11-24 with *E. coli* BW25113, BW25113 Δ*fepA*, and an *E. coli* gut isolate (termed DIEC), isolated from ED01. Images were acquired using a 100× oil immersion objective with a fluorescence exposure time of 429 ms, for all samples. Scale bar: 50 µm; (B) Boxplots display background-corrected, SYTO-normalised azide fluorescence intensities in the three bacterial strains shown in A, measured on a plate well using a plate reader. Each point represents a technical replicate, with three technical replicates and three biological replicates analysed for each *E. coli* strain (*n* = 9); samples are color-coded by biological replicate. Boxes represent the interquartile range (IQR), with horizontal lines indicating the median; whiskers extend to 1.5× the IQR. Jittered points depict within-group variability. Statistical significance was assessed by one-way ANOVA (overall *P*-value shown), followed by pairwise comparisons using the Wilcoxon test with Benjamini-Hochberg correction. Significance levels: **** *P* < 0.0001, *** *P* < 0.001, * *P* < 0.05. *E. coli*: *Escherichia coli*.

The molecular mechanisms of human-targeted drug bioaccumulation by microbes remain poorly understood. To investigate the determinants of entacapone bioaccumulation, we used alkyne-labelled derivatives and *E. coli* as a model system, given its ability to bioaccumulate entacapone and the availability of comprehensive mutant libraries for genetic analysis^[42]^. As entacapone binds to iron in a manner similar to that of siderophores^[2]^, we hypothesised that siderophore transporters might mediate its uptake. To test this hypothesis, we compared the accumulation of entacapone in wild-type *E. coli* BW25113 and a mutant lacking the ferrienterobactin (siderophore) receptor FepA^[43]^. *E. coli* samples were incubated with the alkyne-tagged derivative EB-11-24. *E. coli* is expected to accumulate entacapone to high levels^[2]^, and our preliminary results showed that EB-11-24 produced a strong signal for tracking its bioaccumulation in these cells, without issues of signal intensity (data not shown). Fluorescence quantification revealed a strong signal in the wild-type cells, whereas the ∆*fepA* cells exhibited markedly reduced fluorescence intensity per cell [Figure 6A and B]. This implicates FepA in entacapone uptake. Notably, a gut-isolated *E. coli* strain (DIEC, isolated from ED01) exhibited even higher fluorescence than *E. coli* BW25113, suggesting either enhanced FepA-mediated transport or the existence of alternative uptake mechanisms [Figure 6A and B]. Overall, these results suggest the existence of a FepA-dependent pathway for entacapone internalisation in *E. coli* and emphasise the effectiveness of bioorthogonal labelling strategies for the screening of drug uptake in microbial populations.

## DISCUSSION

Bioorthogonal drug labelling has emerged as a powerful approach for studying drug distribution and toxicity in biological systems with high specificity and minimal interference^[44]^. To date, most applications have centred on identifying host drug targets, optimising delivery, and enabling controlled therapeutic activation^[45]^. Given the increasing recognition of the gut microbiome’s role in drug pharmacokinetics, bioorthogonal labelling holds strong potential to probe drug-microbiome interactions^[11]^. Here, we apply this approach for the first time to monitor drug bioaccumulation within a complex microbial community. A key premise is that these drug derivatives must replicate the native drug’s effects on the microbiota, as changes in drug fate or cellular interactions could invalidate the method^[34]^. Entacapone was an ideal test candidate for this approach, as its effects on microbial communities are well-characterised^[2]^. Entacapone’s microbial activity is primarily mediated by its nitrocatechol ring^[46]^. Tagging functional groups that disrupt the integrity or function of this ring could compromise the utility of labelled compounds for studying entacapone-microbe interactions. For this reason, we positioned the alkyne tags away from the nitrocatechol ring. By comparing the impact of these alkyne derivatives with that of the native entacapone molecule on the microbiome, we were able to confirm that labelled entacapone retained full biological activity.

While the impact of entacapone on gut microbial composition has been studied^[2,39,47,48]^, the extent of its bioaccumulation by the microbiome and how this varies across individuals remains underexplored^[2]^. This is a critical gap, as bioaccumulation can affect both drug efficacy and microbial community dynamics, with potential consequences for the host. We show that entacapone accumulates widely across diverse microbial taxa, with a consistently high percentage of labelled cells in samples from three donors with distinct microbiome compositions. However, not all cells within the communities displayed entacapone signal. This suggests that even within phylogenetically identical groups, some cells accumulate the drug while others do not, potentially as a result of phenotypic heterogeneity affecting microbial activity and, consequently, drug bioaccumulation^[49]^. Indeed, many microbes exhibit significant phenotypic diversity within isogenic strains^[50]^. Additionally, heterogeneous drug accumulation may be caused by varying local drug concentrations in different parts of the sample, for instance, due to barriers to drug diffusion or the presence of different neighbouring microbes^[12]^.

Bioaccumulation of human-targeted drugs by gut bacteria has only recently been reported, with approximately a dozen drugs identified to date^[11,12]^. For most human-targeted drugs, including entacapone, the molecular mechanisms remain poorly understood. However, studies on microbial uptake of other small molecules, such as antibiotics and mycotoxins, offer useful insights^[51,52]^. For instance, the accumulation of certain mycotoxins by gut microbes has been linked to their ability to penetrate the cell wall^[52]^. A parameter that dictates the capacity of a compound to penetrate the bacterial cell wall is its lipophilicity, which increases affinity to lipids and passive passage through the lipid bilayer. Entacapone has been reported to have low lipophilicity^[53]^, and we did not observe any particular accumulation at the cell membrane. Instead, its signal is distributed throughout the cell, suggesting that uptake occurs via active transport rather than membrane affinity. Given entacapone’s iron-binding properties, similar to those of siderophores, we tested whether siderophore transporters mediate its uptake. Suppression of these transporters (FepA) reduced entacapone accumulation but did not eliminate it, indicating alternate transport mechanisms, possibly other iron transporters or broader uptake pathways, are involved. This is consistent with the observation that only a subset of the microbial community produces siderophores^[54,55]^, yet entacapone accumulation is widespread. Drugs can also accumulate intracellularly by binding to specific proteins or cellular structures^[56]^. For example, duloxetine interacts with proteins involved in amino acid metabolism, purine and pyrimidine biosynthesis, and the pentose phosphate pathway in *Clostridium saccharolyticum*^[11]^. Whether entacapone binds intracellular proteins remains unknown, but bioorthogonal labelling is well-suited to address this question, being compatible with advanced imaging techniques such as super-resolution fluorescence microscopy and click-electron microscopy^[57]^. Furthermore, studies with fluoroquinolones show a strong negative correlation between intracellular drug concentration and efflux pump activity^[14]^, suggesting that the interplay between uptake and efflux may be a key determinant of entacapone, and more broadly, drug bioaccumulation.

### Limitations of the study

In summary, we successfully used biorthogonal labelling to track entacapone accumulation in complex faecal microbiome samples, revealing broad uptake across diverse taxa with some phenotypic variability. However, our study has some limitations. This study provides mechanistic insights, but its nature was exploratory, limited to a small cohort of healthy young donors. The microbiota structure of patients with PD differs somewhat from that of healthy controls^[58]^, highlighting the need for larger studies in this population. Mechanistic understanding of entacapone bioaccumulation is still in its early stages. Future studies should include genome-wide screening of complete mutant libraries to identify additional molecular factors that influence drug accumulation. We also did not assess whether microbial bioaccumulation affects drug bioavailability. Since some degree of drug absorption is expected in the colon^[59]^, *in vivo* studies are needed to determine whether microbial uptake in the colon influences systemic drug levels or therapeutic efficacy. Our findings highlight bioorthogonal labelling as a powerful tool to study drug-microbe interactions, providing insights that could help predict or mitigate microbiome-driven side effects and improve personalised therapies.

## DECLARATIONS

### Availability of data and materials

The 16S rRNA sequencing data has been deposited in the National Centre for Biotechnology Information (NCBI) Sequence Read Archive under Bioproject number PRJNA1295500. The databases used were the RDP taxonomy release, the rrnDB-5.9 PantaxaStats dataset, and the SILVA taxonomy (release 138) using the Mothur classifier.

### Copyright

© The Author(s) 2025.
