# Supplementary material for: Taxonomic and mechanistic insights into gut microbiota bioaccumulation of entacapone using bioorthogonal drug labelling
Source: Microbiome Res Rep. 2025 Nov 20;4(4):41. doi: 10.20517/mrr.2025.73 (PMC12719382; doi:10.20517/mrr.2025.73)
Supplement: Supplementary file 1 [file mrr-4-4-41-SupplementaryMaterials.zip › mrr-4-4-41-SupplementaryMaterials/mrr4073-Supplementary Figures.pdf]

Supplementary Materials

Taxonomic and mechanistic insights into gut microbiota bioaccumulation of entacapone using bioorthogonal drug labelling

Linda M. Guantai[1,3], Clementine E. Bavinton[2], Juwairiyah B. Shazzad[1], Sumeet Mahajan[2,3], Sam Thompson[2,3] Fatima C. Pereira[1,3]

[1]School of Biological Sciences, Faculty of Environmental and Life Sciences, University of Southampton, University Road, SO17 1BJ, United Kingdom.
[2]School of Chemistry and Chemical Engineering, Faculty of Engineering and Physical Sciences, University of Southampton, Southampton, SO17 1BJ, United Kingdom.
[3]Institute for Life Sciences, University of Southampton, Southampton SO17 1BJ, United Kingdom.

Correspondence to: Dr. Fatima C. Pereira, School of Biological Sciences, Faculty of Environmental and Life Sciences, University of Southampton, Southampton SO17 1BJ, United Kingdom. E-mail: f.c.pereira@soton.ac.uk

ORCID: Fatima C. Pereira (0000-0002-1288-6481)

This file contains:

Supplementary figures 1-4……………………………………………………… Pages 2 - 5
Supplementary materials and methods, including Supplementary figures 5 to 17…Pages 6-17

Supplementary figures 1-4

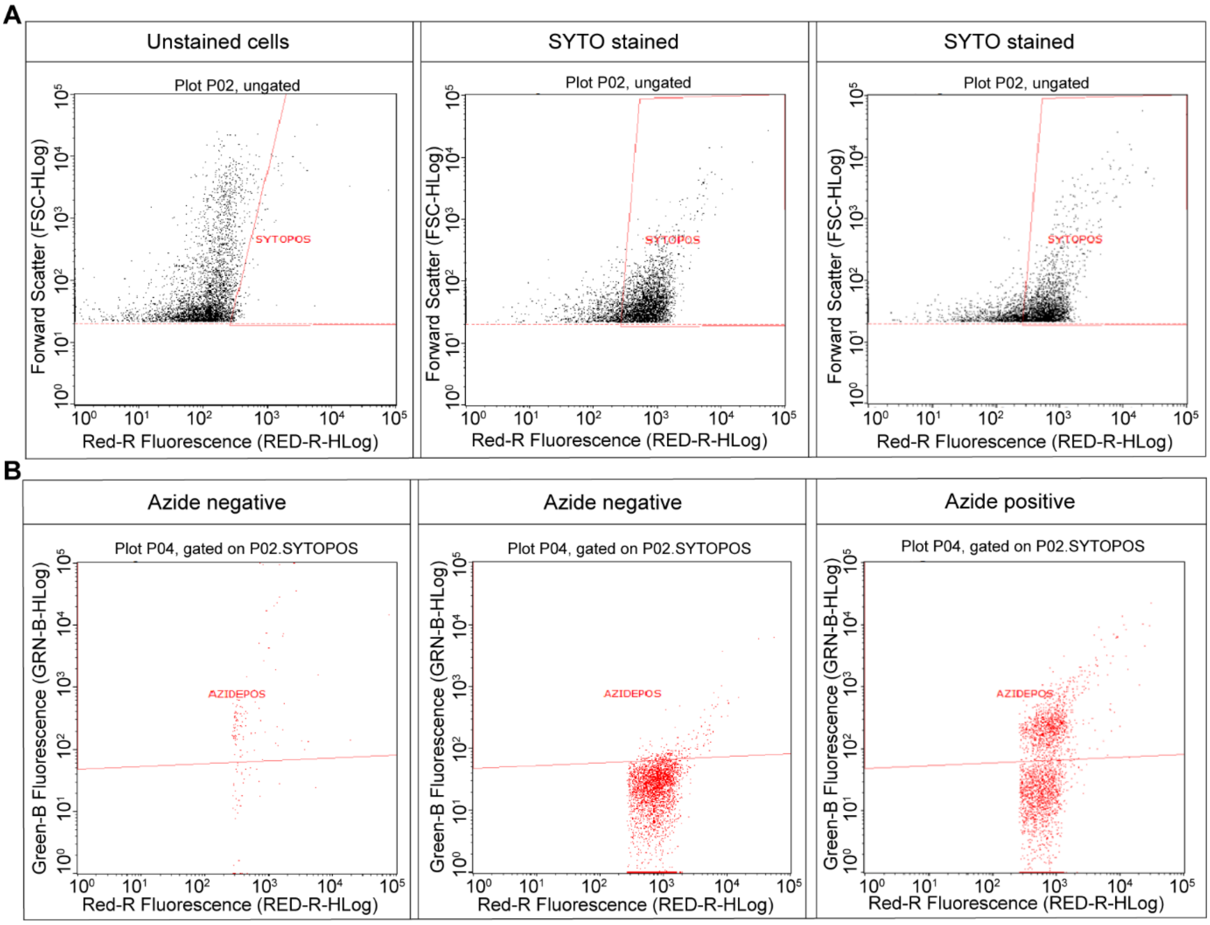

Supplementary Figure 1. Flow cytometry gating strategy for quantifying microbial interactions with fluorescently labelled entacapone derivatives. Flow cytometry was used to distinguish microbial cells interacting with fluorescently labelled entacapone derivatives. (A) SYTO™ Deep Red gating (Forward scatter vs Red-R fluorescence) using unstained and stained controls to identify microbial cells. (B) Alexa Fluor™ 488 azide gating within SYTO™-positive cells (Green-B fluorescence vs Red-R fluorescence) using azide-negative and azide-positive controls to detect cells incorporating AF488-labelled EB-11-38.

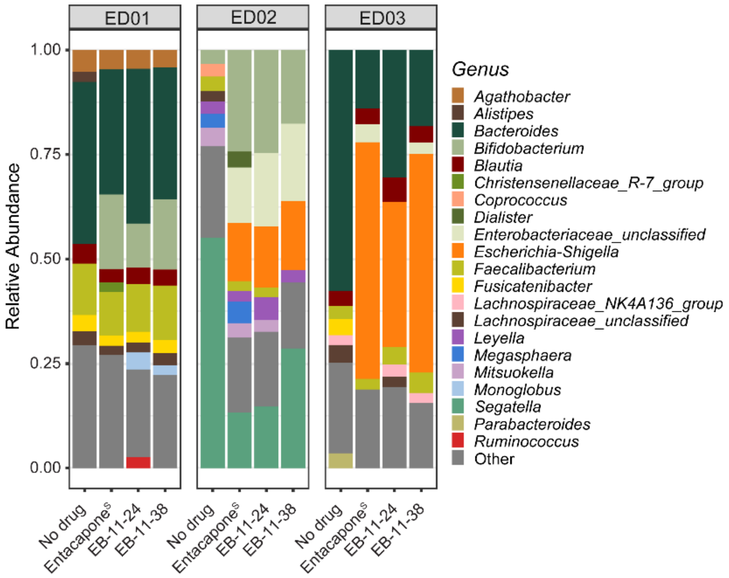

Supplementary Figure 2. Relative abundances of genera assessed using 16S rRNA gene amplicon sequencing in faecal samples treated with EB-11-24, EB-11-38, entacapone^{S}, and the no drug control, separated per donor. Taxa with a relative abundance below 1% were grouped as “Other”.

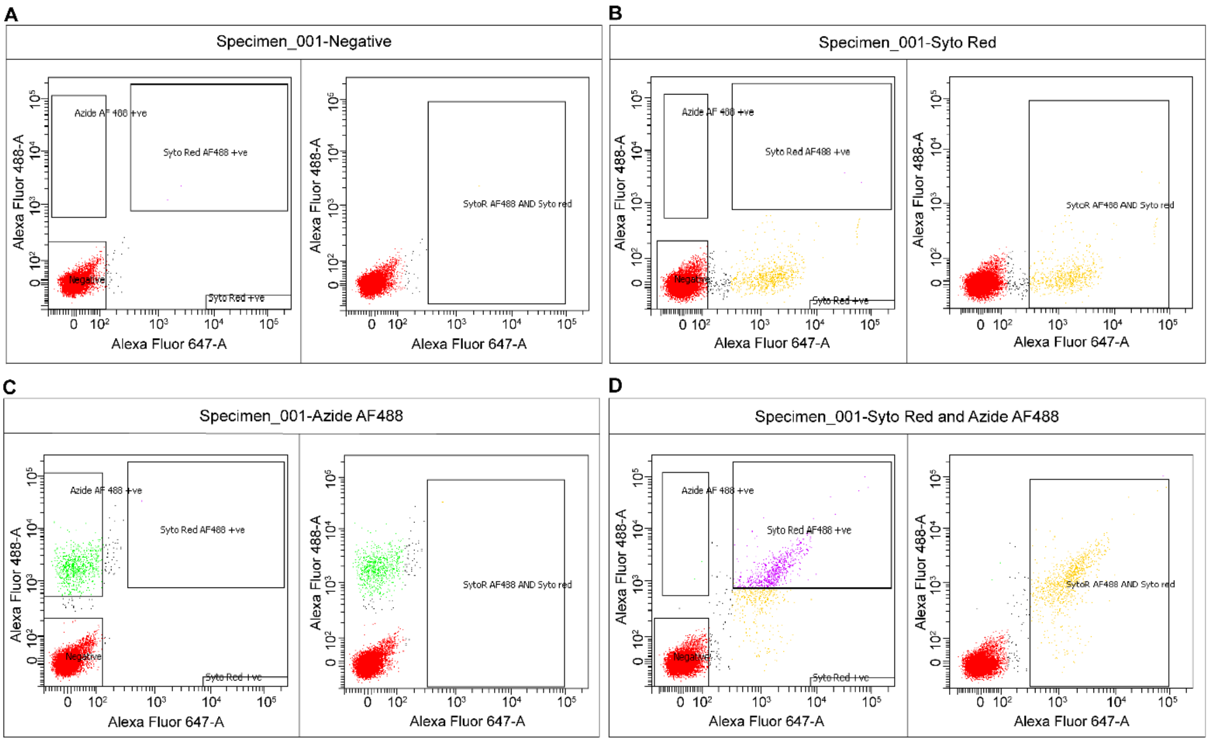

Supplementary Figure 3. Flow Cytometry gating to sort cell events interacting and not interacting with fluorescently labelled EB-11-38. (A) Negative, unlabelled control (events coloured red); (B) Gating of SYTO^{TM} Deep Red positive cells (events coloured yellow) to distinguish cells from debris and background noise; (C) Gating of Alexa Fluor^{TM} 488 azide-

positive, SYTO unstained cells (AF488, events coloured green) to discriminate AZIDE-positive cells; (D) Gating of Alexa Fluor™ 488 azide-positive and SYTO™ Deep Red stained cells (events coloured purple) to discriminate AZIDE and SYTO-positive cells.

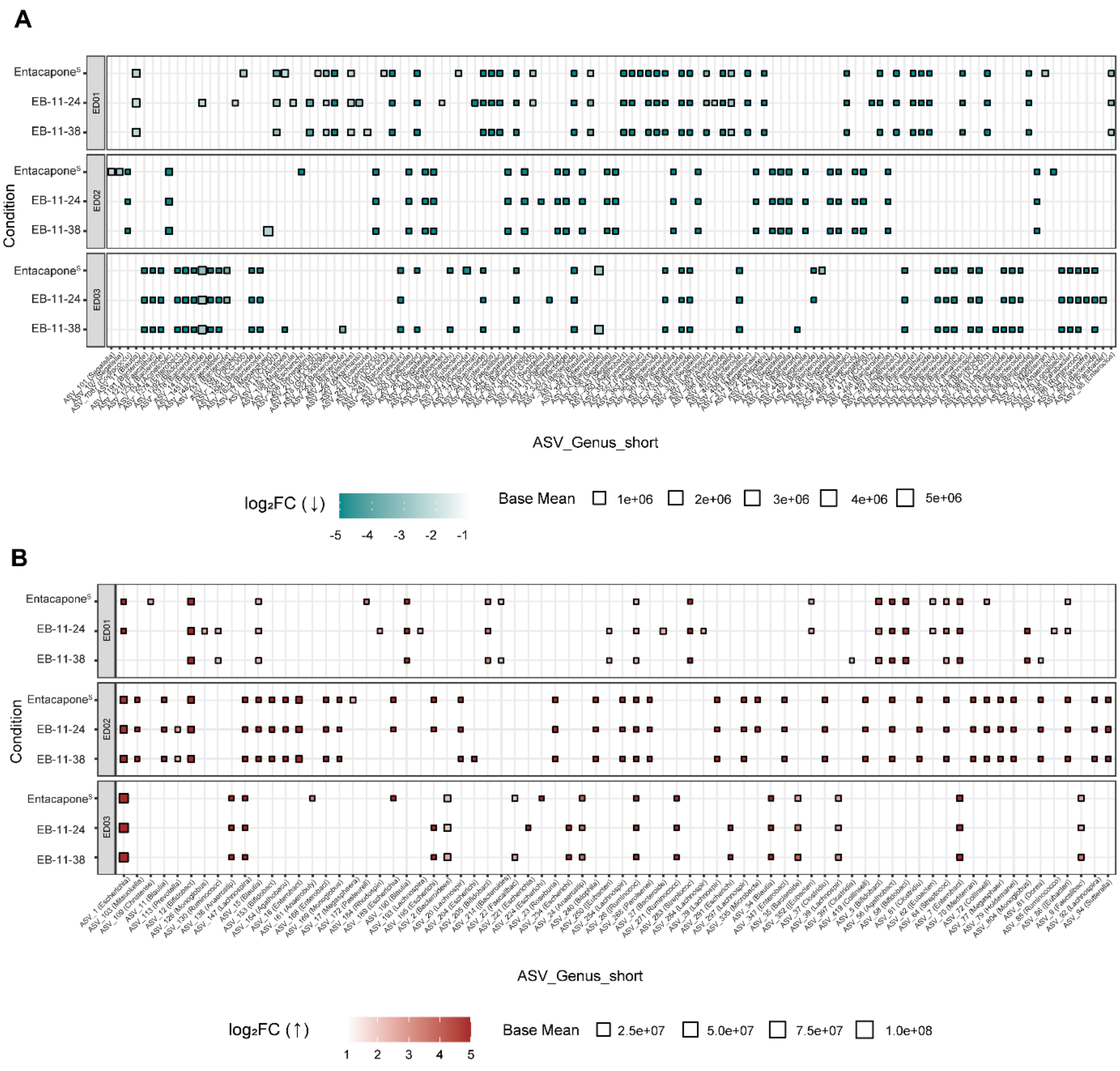

Supplementary Figure 4. ASVs affected by entacapone and its derivatives. (A) Heatmap depicting differentially abundant ASVs across treatment conditions (entacapone^{S}, EB-11-24, EB-11-38) for each donor, depicted in rows. The x-axis represents ASVs that were significantly decreased under each treatment compared to no-drug ($log_2FC<-1$, $p_{adjusted}<0.05$, DESeq2 analysis), while the y-axis represents the respective treatment; (B) ASVs that were significantly increased in abundance under each treatment condition relative to the no-drug control ($log_2FC > 1$, $P_{adjusted} < 0.05$, DESeq2 analysis), across all donors. Point size indicates

the average abundance (base mean), and colour represents the Log2 fold change (log_2FC) with values capped at -5 or 5 to enhance visual clarity.

Supplementary Materials and Methods including Supplementary Figures 5 to 17

1. Fluorescence microscopy

Clicked samples (10 μL) were spotted onto a glass slide and allowed to dry at 46°C. Samples were stained with 4',6-diamidino-2-phenylindole (DAPI) for 10 min using 20 μL of 1 $\mu g \cdot mL^{-1}$ DAPI (Sigma-Aldrich) per well, followed by a single wash in ice-cold MilliQ water. Slides were subsequently dried with pressurised air, and a drop of CitiFluor AF1 anti-fading agent was added before applying the coverslip. Images were acquired on an Olympus IX83 inverted microscope, using a 100× oil objective. Images were captured with cellSens software v4.2 (Evident Scientific) and were analysed using FIJI version 2.9.0[1].

2. Determination of AF488-positive cells by flow cytometry

To determine the percentage of microbial cells interacting with EB-11-24 and EB-11-38 [Supplementary Table 6], a Guava easyCyte™ cytometer was used. A minimum of 50,000 events were recorded per analysed sample. Gating on SYTO™ Deep Red dye was performed using SYTO™ Deep Red negative control (unstained cells) and positive control (SYTO™ stained cells), creating a gate for the detection of microbial cells based on nucleic acid staining, using a 638nm laser [Supplementary Figure 1A]. Alexa Fluor™ (AF) 488 azide positive and negative controls were then used to establish a gate for azide labelled cells (488nm laser) within SYTO™ Deep Red positive cells, determining the percentage of active cells incorporating AF488-azide [Supplementary Figure 1B]. Using InCyte software, the sample was analysed by dividing the count of azide and SYTO™ Deep Red positive events by the total SYTO™ Deep Red positive counts. All analyses were conducted within 6 h of sample preparation.

3. Extraction of DNA from sorted cells

Nucleic acids were extracted using the QIAamp DNA Kit (Qiagen, UK). For each sample, one tube contained SYTO™ Deep Red-stained cells, while the other included both azide-positive and SYTO™ Deep Red stained cells. Sorted cells were treated with 200 μL of enzymatic lysis buffer directly on the FACS collection tube (comprising 20 mM Tris-Cl, 2 mM sodium EDTA, 1.2% Triton, and 20 mg/mL lysozyme added just before use) and incubated at 37 °C for 30 min while shaking at 200 rpm. Subsequently, 15 μL of proteinase K was added, and the mixture was vortexed. The remaining steps followed the manufacturer's protocol.

4. 16S rRNA gene amplicon sequencing of sorted cells

16S rRNA gene amplification and sequencing of the bacterial DNA were carried out as described above for faecal samples incubated with drugs. After the removal of contaminants, 1,605 taxa were retained. The average read count per sample was 53,710 ± 12,112. The sample coverage was above 99% for all samples [Supplementary Tables 10].

5. Assessment of entacapone accumulation via fluorescence measurements

E. coli strains, including a laboratory-grown strain (D1EC), and wild-type *E. coli* BW25113 and a BW25113 *fepA* mutant, were cultured and exposed to EB-11-24. A no-drug treatment served as the control. Following 24 h of anaerobic incubation in M9 minimal medium, the samples were pelleted and washed in 1x phosphate-buffered saline (PBS). Cells were fixed with 3% paraformaldehyde (PFA) for 2 h at 4 °C. Fixed cells were washed twice with 1 mL of PBS and stored in a 1:1 PBS: ethanol solution (v/v) at -20 °C. Samples were subjected to a click chemistry reaction and stained with SYTO™ Deep Red, as previously described. Fluorescence measurements were performed using a FLUOstar OPTIMA plate reader (BMG LABTECH, UK). SYTO™ Deep Red fluorescence was measured using excitation at 605 ± 15 nm and emission at 670 ± 25 nm. Alexa Fluor™ 488 (azide) was measured using excitation at 488 ± 7 nm and emission at 535 ± 15 nm. Assays were conducted in COSTAR 96-well half-area microplates with 20 flashes per well and a focal height of 5.7 mm. Samples were shaken for 10 seconds at 300 rpm (orbital) before each reading cycle. A test run was conducted to assess cross-well signal bleeding [Supplementary Table 11].

6. Chemical synthesis of entacapone derivatives

6.1 General methods for chemical synthesis

Any anhydrous solvents were used as supplied by major chemical suppliers (Sigma-Aldrich, Merck, Acros Organics, Fisher Scientific) with SureSeal™ or similar bottles. Other solvents were used directly from the bottle. Aqueous solutions were saturated unless specified otherwise. All reagents were used directly as supplied by major chemical suppliers without further purification.

Chromatography

Flash column chromatography was carried out using Geduran® Si 60 silica gel, and analytical thin-layer chromatography was carried out using Merck Kieselgel 60 F_{254}

fluorescent-treated silica. These plates were visualised under ultraviolet (UV) light (254 and 260 nm), followed by staining with potassium permanganate solution.

NMR spectroscopy

^{1}H and ^{13}C nuclear magnetic resonance (NMR) spectra were recorded using a Bruker-SpectroSpin 400 or 500 MHz spectrometer with TopSpin™ software. All chemical shifts (δ) are quoted in ppm using a residual solvent peak ($CDCl_3$, MeOH-d_4, or dimethyl sulfoxide (DMSO)-d_6) as the internal standard. Coupling constants (J) are given in Hertz (Hz).

The ^{1}H NMR spectra are reported as: δ_H (spectrometer frequency / MHz, solvent): δ / ppm (integration, multiplicity, coupling constant J / Hz, where appropriate, to the nearest 0.1 Hz). Multiplicity has been abbreviated as follows: s = singlet, d = doublet, t = triplet, sept = septet, m = multiplet. The ^{13}C NMR spectra are reported as: δ_C (spectrometer frequency / MHz): δ / ppm.

All NMR data was recorded at 298 K unless otherwise stated [Supplementary Figures 10-14].

IR Spectroscopy

Infrared (IR) spectra were recorded using a ThermoFisher Scientific Nicolet iS5 spectrometer. A total of 16 scans were collected at a resolution of 4 cm^{-1}. Selected maximum absorbances (ν_{max} / cm^{-1}) are given for the most intense peaks.

Mass Spectrometry

Ultrahigh performance liquid chromatography-mass spectrometry (UHPLC-MS) was performed using a Waters (Manchester, UK) Acquity TQD mass tandem quadrupole mass spectrometer (Electrospray Ionisation (ESI), reversed phase.) High-resolution ESI mass spectra were recorded using a MaXis (Bruker Daltonics, Bremen, Germany) time of flight (TOF) mass spectrometer or a solariX (Bruker Daltonics, Bremen, Germany) mass spectrometer equipped with a 4.7 T magnet and Fourier transform ion cyclotron resonance (FT-ICR) cell. Gas Chromatography-mass spectrometry (GC-MS), electron ionisation (EI) used on a Thermo (Hemel Hempstead, UK) Trace GC-MS single quadrupole mass spectrometer [Supplementary Figures 14-17].

Melting point

Melting points were recorded using a Stuart (Bibby Scientific Limited) SMP20 machine and are reported uncorrected in °C.

Synthesis of (E)-2-Cyano-3-(3,4-dihydroxy-5-nitrophenyl)-N, N-diethylacrylamide (Entacapone[s])

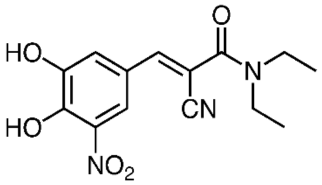

Supplementary Figure 5. Chemical structure of (E)-2-Cyano-3-(3,4-dihydroxy-5-nitrophenyl)-N, N-diethylacrylamide (Entacapone[s])

Prepared according to US Patent 20100234632A1[2], 3,4-dihydroxy-5-nitrobenzaldehyde (CAS 116313-85-0, 3.00 g, 16.4 mmol) and diethylcyanoacetamide (CAS 26391-06-0, 2.3 mL, 16.4 mmol) were dissolved in toluene: cyclohexane (30 mL, 1 : 1 *v:v*) at room temperature (r.t), followed by the addition of piperidine (0.16 mL, 1.64 mmol). The mixture was heated to reflux at 120 °C, with water removed azeotropically using Dean-Stark apparatus. After 4 h, glacial acetic acid (6.0 mL) was added, followed by cooling to r.t. After stirring, the resulting precipitate was collected by filtration and washed with toluene and water. The residue was dried *in vacuo* to afford Entacapone[s] as an orange-brown solid (3.91 g, 12.8 mmol, 78 %) [Supplementary Figures 5 and 10].

δ_H (400 MHz, DMSO-d_6) 10.90 (2H, br), 7.93 (1H, d, *J* 2.1), 7.75 (1H, d, *J* 2.1), 7.63 (1H, s), 3.41 (4H, q, *J* 5.8), 1.15 (6H, t, *J* 6.1); δ_C (126 MHz, DMSO-d_6) 162.8, 148.2, 147.4, 145.3, 137.3, 122.7, 118.6, 117.8, 116.4, 104.8, 43.2, 43.3, 13.7, 12.8; MP 155-157.

Synthesis of *2-Cyano-*N*-(prop-2-yn-1-yl)acetamide (EB-11-23)*

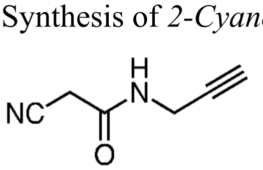

Supplementary Figure 6. Chemical structure of 2-Cyano-N-(prop-2-yn-1-yl) acetamide (EB-11-23)

According to a literature procedure[3] ethyl cyanoacetate (5.0 mL, 46.9 mmol) and dipropargylamine (0.5 mL, 4.83 mmol) were heated at 40 ˚C for 6 h, until t.l.c indicated

consumption of starting material. The orange-red solid in the reaction mixture was recrystallised from MeOH, and washed with CH_2Cl_2, to afford EB-11-23 as a pale-yellow solid (791 mg, 6.48 mmol, 14 %) [Supplementary Figures 6 and 11].

δ_H (400 MHz, DMSO-*d6*) 8.69 (1H, s), 3.90-3.88 (2H, m), 3.65 (2H, s), 3.17 (1H, t, *J* 2.5); δ_C (126 MHz, DMSO-*d6*) 162.3, 116.1, 80.5, 73.7, 28.7, 25.3; MP 108-110.

Synthesis of 2-Cyano-*N*, *N*-di(prop-2-yn-1-yl)acetamide (EB-11-33)

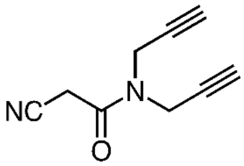

Supplementary Figure 7. Chemical structure *of* 2-Cyano-*N*, *N*-di(prop-2-yn-1-yl) acetamide (EB-11-33).

According to a literature procedure[4], cyanoacetic acid (343 mg, 4.03 mmol) and hydroxybenzotriazole (545 mg, 4.03 mmol) were dissolved in CH_2Cl_2 (30 mL), and dipropargylamine (0.5 mL, 4.83 mmol) was added. The mixture was stirred at r.t for 30 min, before *N, N'*-dicyclohexylcarbodiimide (1.08 g, 5.24 mmol) was added. The mixture was refluxed with vigorous stirring for 16 h until t.l.c indicated complete consumption of the starting material. The reaction mixture was diluted with CH_2Cl_2 (15 mL), washed with saturated ammonium chloride (2 x 15 mL), and the combined organic layers were dried ($MgSO_4$), filtered, and concentrated *in vacuo* to give a pale orange residue. Purification by flash column chromatography (SiO_2, 5 % diethyl ether: CH_2Cl_2) afforded EB-11-33 as a pale-yellow oil (612 mg, 3.82 mmol, 95 %) [Supplementary Figure 7, Supplementary Figure 12, Supplementary Figure 15].

δ_H (400 MHz, MeOH-*d4*) 4.84 (2H, s), 4.31 (2H d, *J* 2.4), 4.28 (2H, d, *J* 2.4), 2.90 (1H, t, *J* 2.4), 2.72 (1H, t, *J* 2.4); δ_C (126 MHz, MeOH-*d4*) 164.6, 115.4, 78.3, 78.0, 75.4, 74.2, 37.7, 35.9, 25.3; ν_{max} 3284, 2955, 2261, 2123, 1660, 1437, 1212, 1174, 958, 673; High resolution mass spectrometry (HRMS) (GC-EI) found 160.0631, $C_9H_8N_2O$ $[M]^+$ requires 160.0631.
Synthesis of (*E*)-2-Cyano-3-(3,4-dihydroxy-5-nitrophenyl)-*N*-(prop-2-yn-1-yl)acrylamide (EB-11-24)

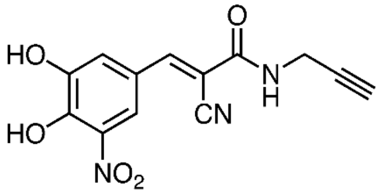

Supplementary Figure 8. (*E*)-2-Cyano-3-(3,4-dihydroxy-5-nitrophenyl)-*N*-(prop-2-yn-1-yl) acrylamide (EB-11-24)

Based on a modification of US patent 20100234632A1[2],3,4-dihydroxy-5-nitrobenzaldehyde (153 mg, 0.835 mmol) and cyanoacetamide EB-11-23 (102 mg, 0.835 mmol) were dissolved in toluene: cyclohexane (1.5 mL, 1: 1 *v:v*) at r.t., followed by the addition of piperidine (8.0 mL, 0.0835 mmol). The mixture was heated to reflux in an oil bath at 120 °C, with water removed azeotropically using Dean-Stark apparatus. After 4 h, glacial acetic acid (0.3 mL) was added, followed by cooling to r.t. After stirring, the resulting precipitate was collected by filtration and washed with toluene and water. The residue was dried *in vacuo* to afford EB-11-24 as a yellow-orange solid (215 mg, 0.749 mmol, 90 %) [Supplementary Figure 8, Supplementary Figure 13, Supplementary Figure 16].

δ_H (500 MHz, DMSO-d_6) 10.7 (2H, br), 8.75 (1H, t, *J* 5.5), 8.02 (1H, s), 7.94 (1H, d, *J* 2.3), 7.71 (1H, d, *J* 2.3), 3.99 (1H, d, *J* 2.5), 3.98 (1H, d, *J* 2.5), 3.15 (1H, t, *J* 2.5); δ_C (126 MHz, DMSO-d_6) 163.5, 150.2, 149.6, 145.6, 124.2, 123.8, 117.1, 115.8, 115.6, 101.1, 80.8, 73.3, 43.9; ν_{max} 3414, 3254, 3095, 2981, 2219, 2130, 1653, 1541, 1508, 1315, 1212, 1142, 876, 629. HRMS (ESI, +VE) found 288.0618, $C_{13}H_9N_3O_5$ $[M+H]^+$ requires 288.0615; MP 210 – 212.

Synthesis of (*E*)-2-Cyano-3-(3,4-dihydroxy-5-nitrophenyl)-*N*,*N*-di(prop-2-yn-1-yl)acrylamide (EB-11-38)

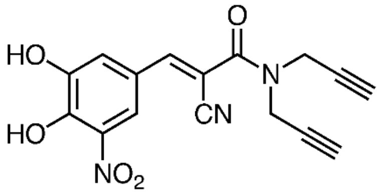

Supplementary Figure 9. Chemical structure of (E)-2-Cyano-3-(3,4-dihydroxy-5-nitrophenyl)-N, N-di(prop-2-yn-1-yl) acrylamide (EB-11-38)

Based on a modification of US Patent 20100234632A1[2], 3,4-dihydroxy-5-nitrobenzaldehyde (280 mg, 1.53 mmol) and cyanoacetamide EB-11-33 (245 mg, 1.53 mmol) were dissolved in toluene: cyclohexane (2.8 mL, 1 : 1 *v:v*) at r.t, followed by the addition of piperidine (0.02 mL, 0.153 mmol). The mixture was heated to reflux in an oil bath at 120 °C, with water removed azeotropically using Dean-Stark apparatus. After 4 h, glacial acetic acid (0.5 mL) was added, followed by cooling to r.t. After stirring, the resulting precipitate was collected by filtration and washed with toluene and water. The solid was discarded and the mother liquor was left to stand overnight, and a yellow-orange solid was collected by gravity filtration. The solid was dried under vacuum to afford EB-11-38 as a yellow-orange solid (26.0 mg, 0.0799 mmol, 5 %) [Supplementary Figure 9, Supplementary Figure 14, Supplementary Figure 17].

δ_H (400 MHz, DMSO-d_6) 10.94 (2H, br), 7.93 (1H, d, *J* 2.1), 7.77 (1H, d, *J* 2.2), 7.73 (1H, s), 4.33 (4H, s), 3.40 (2H, s); δ_C (100 MHz, DMSO-d_6) 163.6, 148.4, 148.1, 145.5, 137.2, 122.2, 119.1, 117.8, 115.7, 103.1, 78.2, 75.9, 41.5; ν_{max} 3288, 3066, 2981, 2359, 2213, 1635, 1539, 1435, 1313, 1234, 1139, 949, 766, 639; HRMS (ESI, +VE) found 326.0779, $C_{16}H_{11}N_3O_5$ $[M+H]^+$ requires 326.0771; MP 155 - 157.

6.2 NMR spectra

Spectra of (*E*)-2-Cyano-3-(3,4-dihydroxy-5-nitrophenyl)-*N*, *N*-diethylacrylamide (Entacapone[s])

^{1}H NMR, 400 MHz, DMSO-d_6

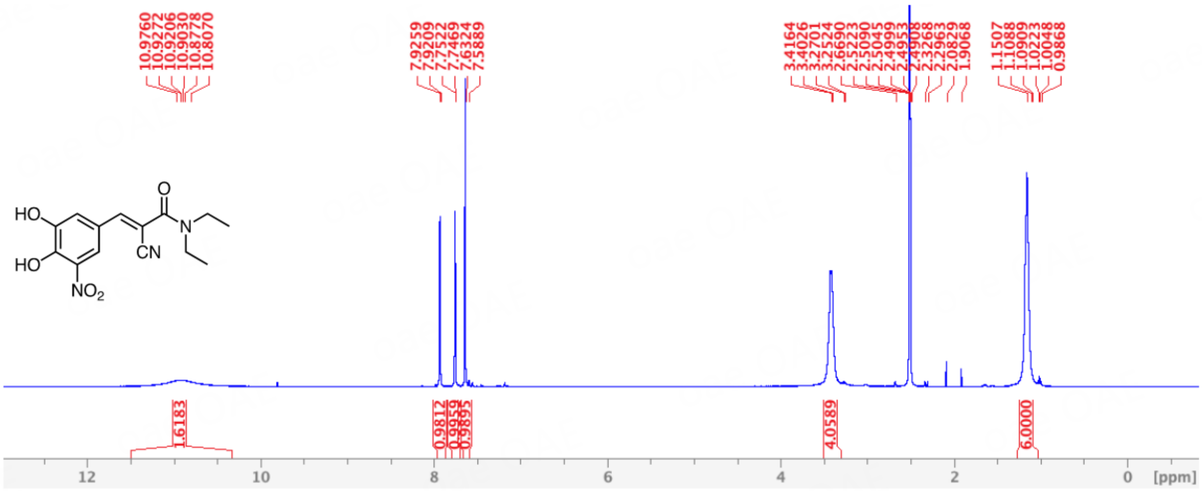

^{13}C NMR, 126 MHz, DMSO-d_6

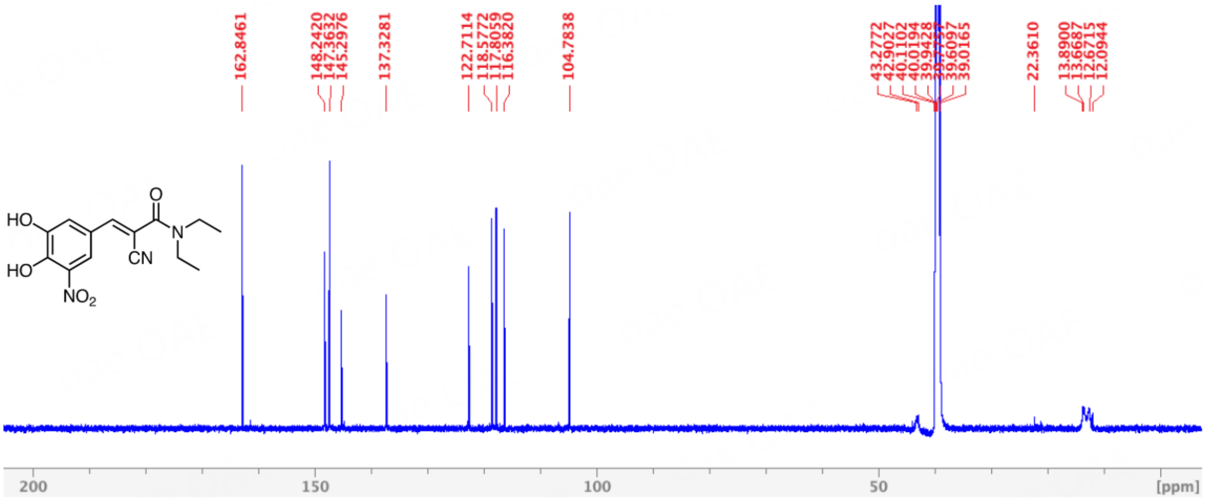

Supplementary Figure 10. ^{1}H (400 MHz) and ^{13}C (126 MHz) NMR spectra of Entacapone[S].

Spectra of 2-Cyano-*N*-(prop-2-yn-1-yl)acetamide (EB-11-23)

^{1}H NMR, 400 MHz, DMSO-d_6

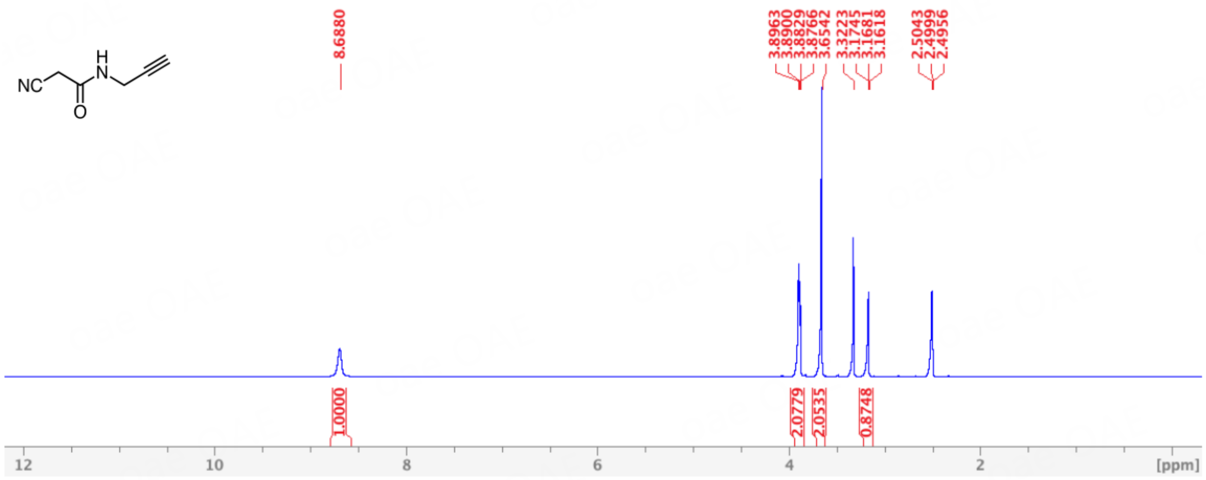

^{13}C NMR, 126 MHz, DMSO-d_6

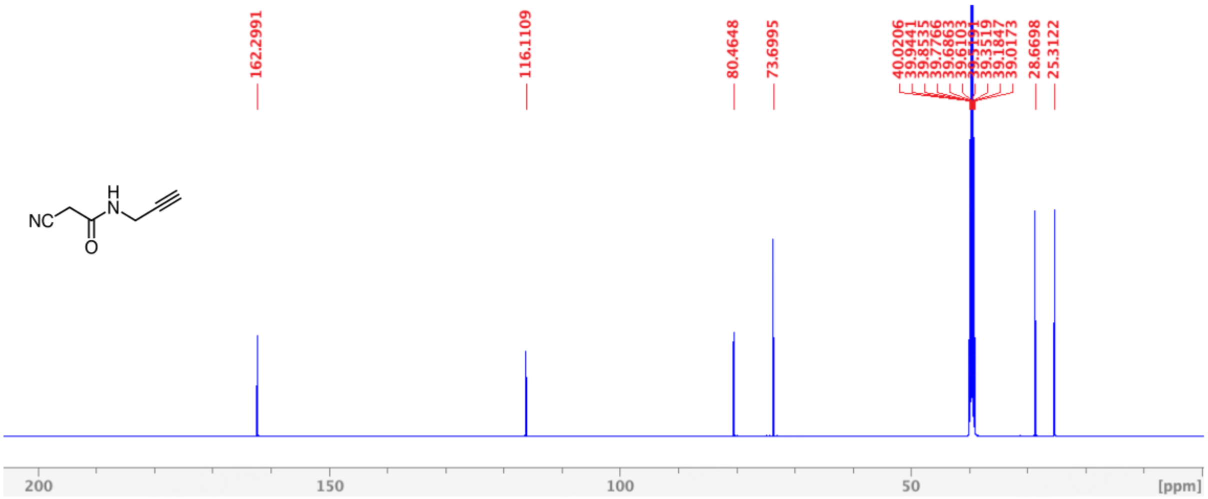

Supplementary Figure 11. ^{1}H (400 MHz) and ^{13}C (126 MHz) NMR spectra of EB-11-23.

Spectra of 2-Cyano-*N*,*N*-di(prop-2-yn-1-yl)acetamide (EB-11-33)

^{1}H NMR, 500 MHz, MeOH-d_4

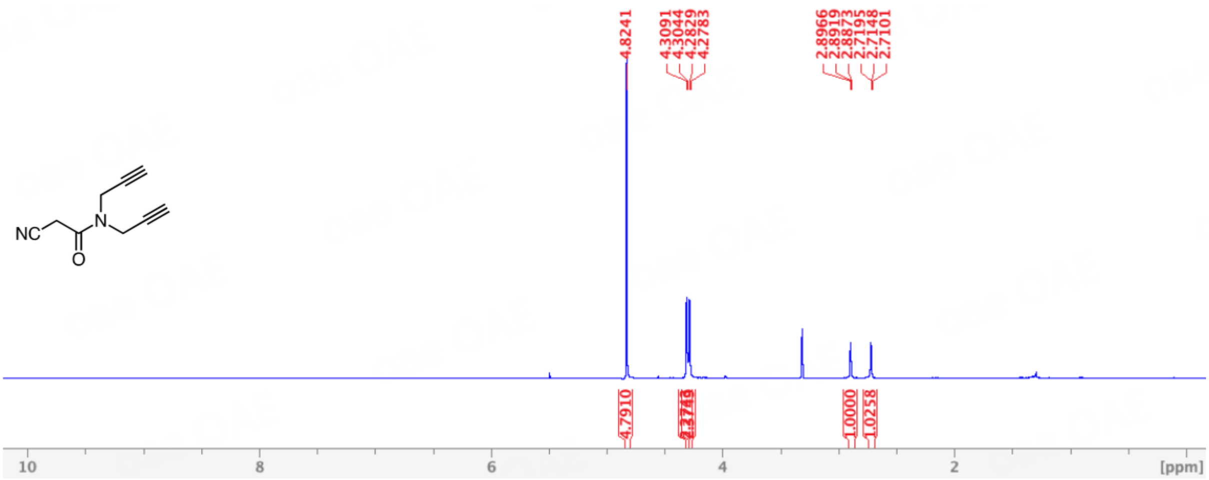

^{13}C NMR, 126 MHz, MeOH-d_4

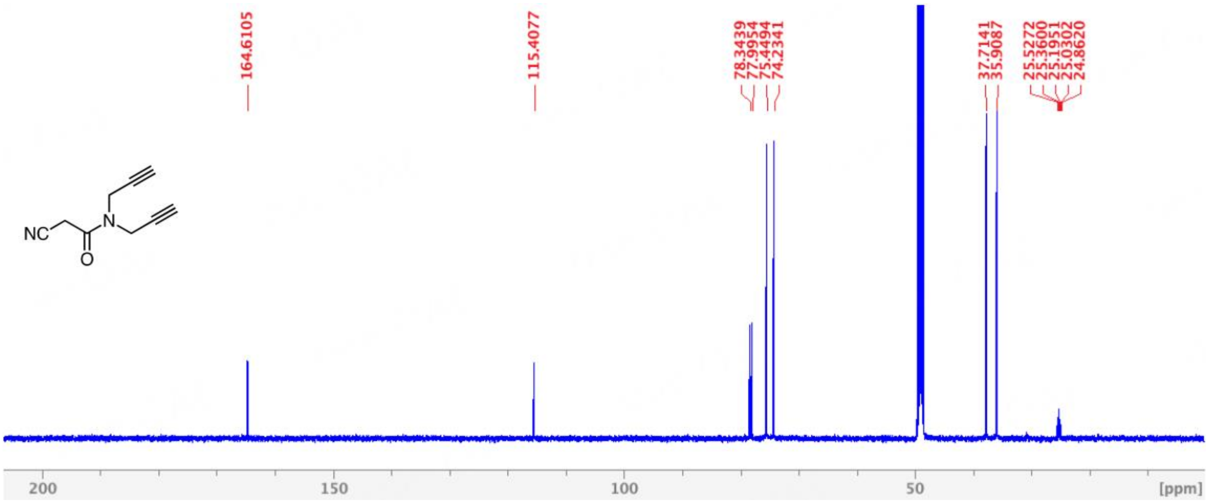

Supplementary Figure 12. ^{1}H (500 MHz) and ^{13}C (126 MHz) NMR spectra of EB-11-33

Spectra of (*E*)-2-Cyano-3-(3,4-dihydroxy-5-nitrophenyl)-*N*-(prop-2-yn-1-yl)acrylamide (EB-11-24)

^{1}H NMR, 500 MHz, DMSO-d_6

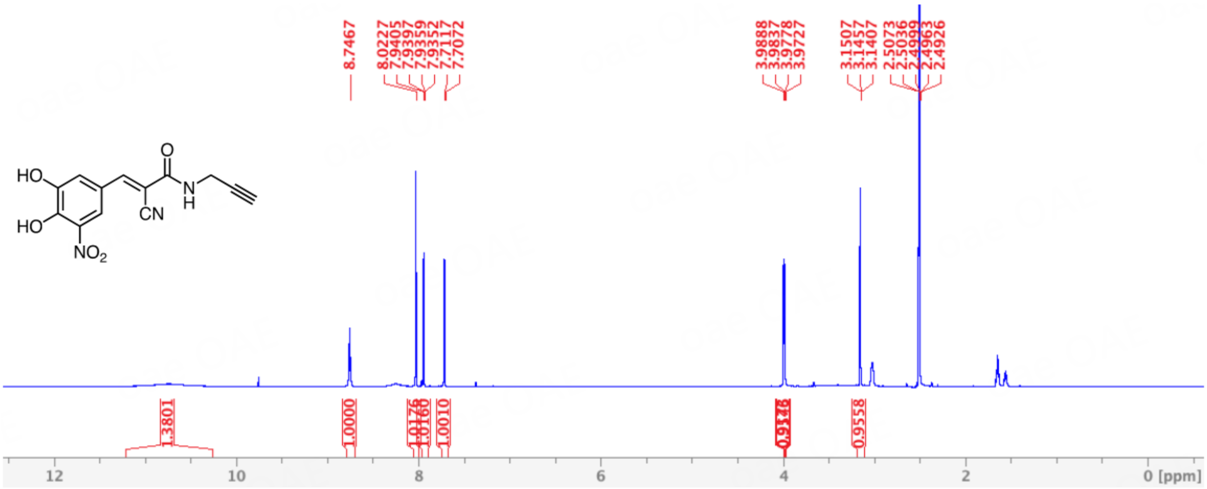

^{13}C NMR, 100 MHz, DMSO-d_6

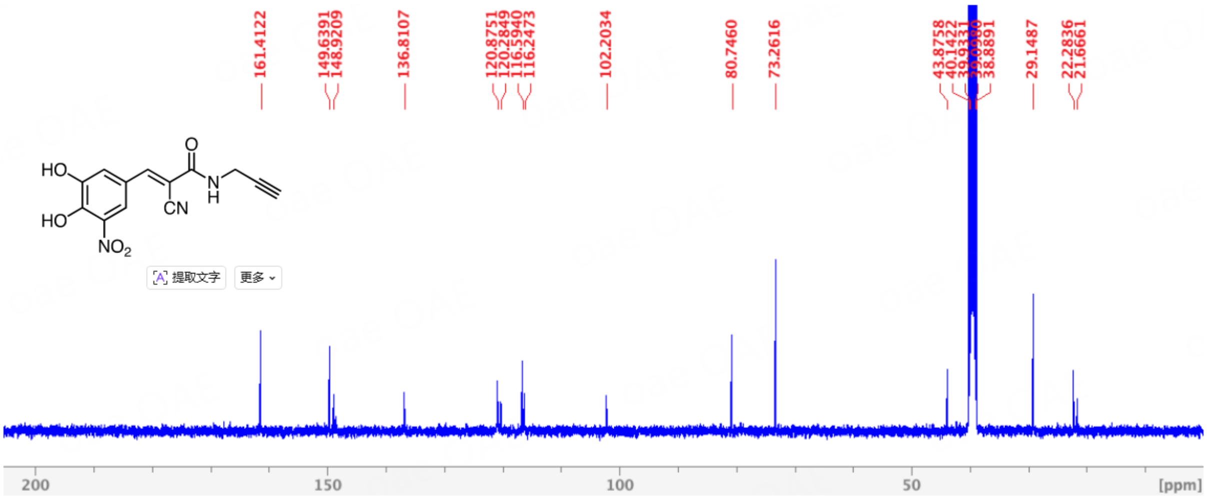

Supplementary Figure 13. ^{1}H (500 MHz) and ^{13}C (100 MHz) NMR spectra of EB-11-24

Spectra of (*E*)-2-Cyano-3-(3,4-dihydroxy-5-nitrophenyl)-*N*,*N*-di(prop-2-yn-1-yl)acrylamide (EB-11-38)

^{1}H NMR, 400 MHz, DMSO-d_6

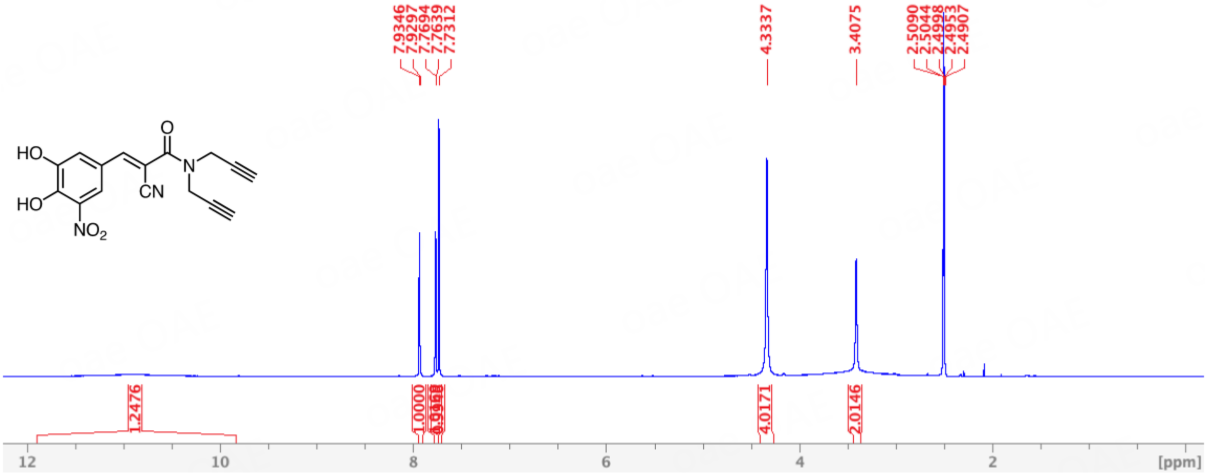

^{13}C NMR, 100 MHz, DMSO-d_6

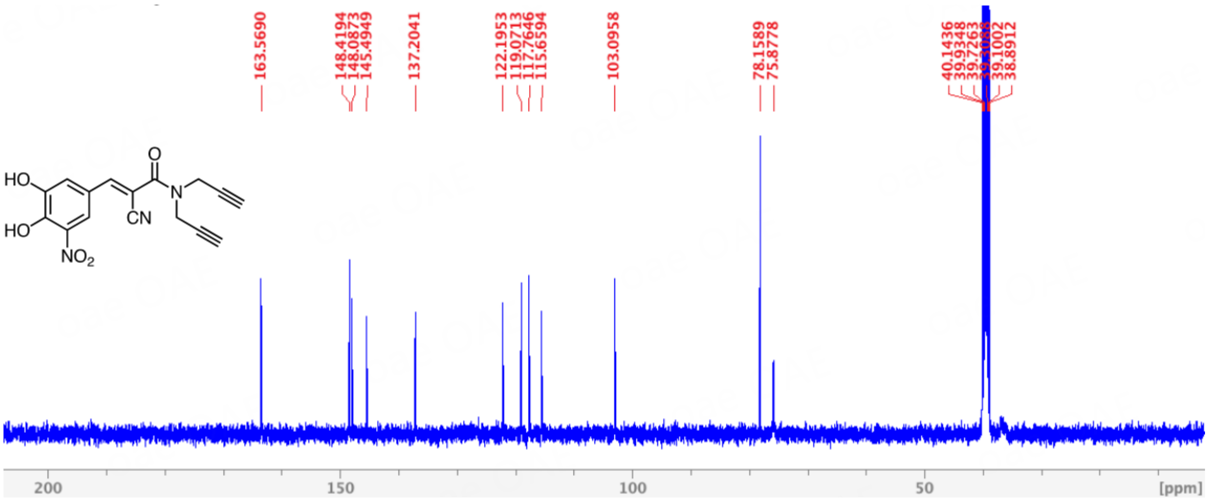

Supplementary Figure 14. ^{1}H (400 MHz) and ^{13}C (100 MHz) NMR spectra of EB-11-38.

HRMS spectra

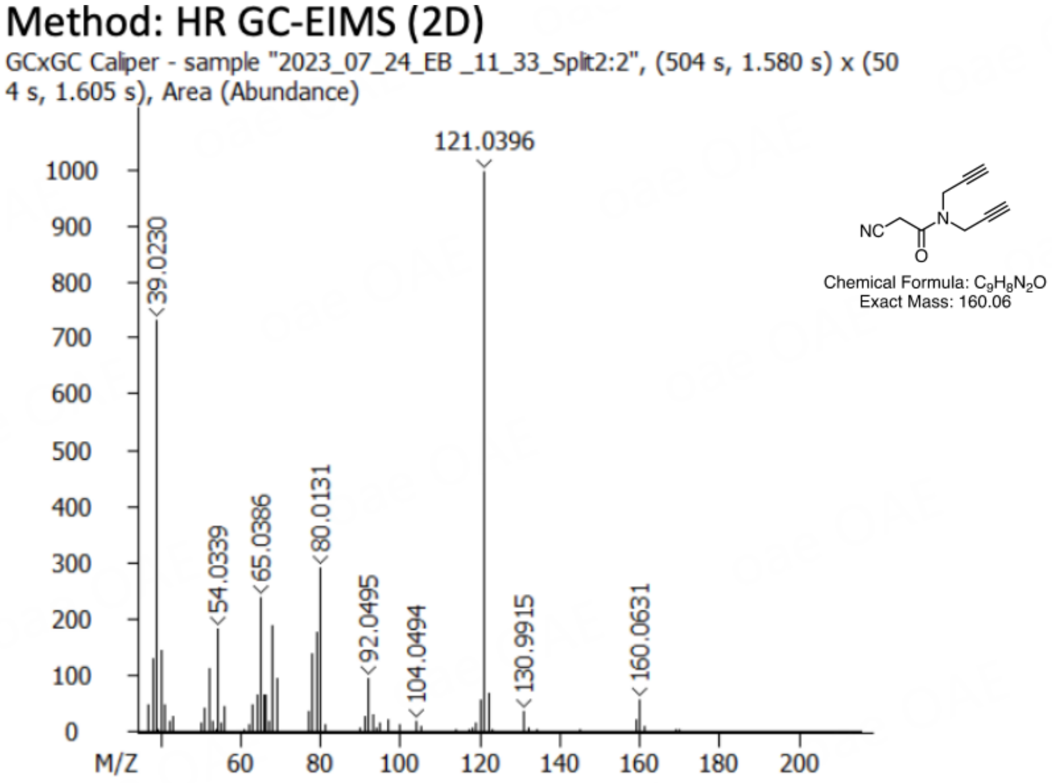

Ion Measured (m/z)	Ion Expected (m/z)	Formula	Error (ppm)	Abundance
**160.0631**	160.0631	$C_9H_8N_2O$	0.09	7
**121.0396**	121.0396	$C_6H_5N_2O$	0.32	100

Supplementary Figure 15. GCMS (EI) of 2-cyano-N, N-di(prop-2-yn-1-yl) acetamide (EB-11-33)

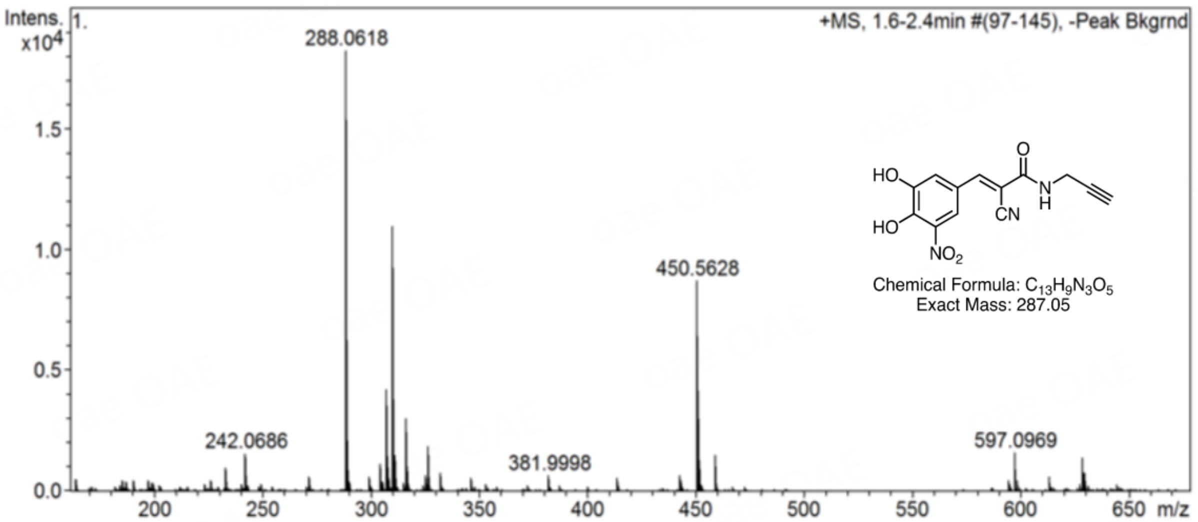

Meas. m/z	Formula	m/z	err [ppm]	err [mDa]	# Sigma	mSigma	rdb	e⁻ Conf	N-Rule
288.0618	C 13 H 10 N 3 O 5	288.0615	-0.9	-0.3	1	9.5	10.5	even	ok
310.0437	C 13 H 9 N 3 Na O 5	310.0434	-0.8	-0.3	1	9.2	10.5	even	ok
	C 11 H 4 N 9 O 3	310.0432	-1.8	-0.5	2	9.3	14.5	even	ok

Supplementary Figure 16. LCMS (ESI+) of (E)-2-Cyano-3-(3,4-dihydroxy-5-nitrophenyl)-N-(prop-2-yn-1-yl) acrylamide (EB-11-24)

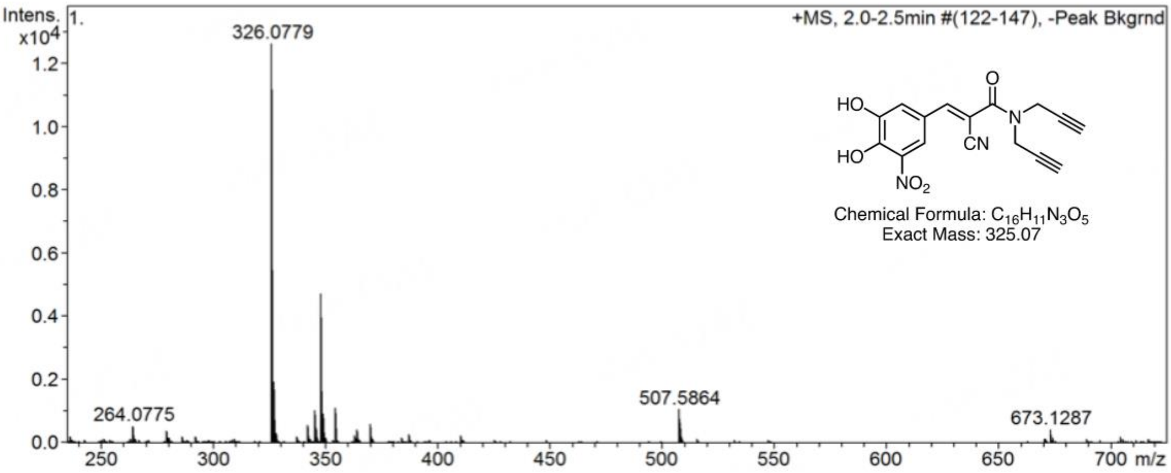

Meas. m/z	Formula	m/z	err [ppm]	err [mDa]	# Sigma	mSigma	rdb	e⁻ Conf	N-Rule
326.0779	C 16 H 12 N 3 O 5	326.0771	-2.3	-0.7	1	18.1	12.5	even	ok
	C 19 H 13 N Na O 3	326.0788	2.7	0.9	2	32.5	13.5	even	ok
	C 17 H 8 N 7 O	326.0785	1.8	0.6	3	32.8	17.5	even	ok
348.0598	C 16 H 11 N 3 Na O 5	348.0591	-2.0	-0.7	1	6.1	12.5	even	ok
	C 14 H 6 N 9 O 3	348.0588	-2.8	-1.0	2	7.6	16.5	even	ok
	C 17 H 7 N 7 Na O	348.0604	1.9	0.6	3	11.8	17.5	even	ok

Supplementary Figure 17. LCMS (ESI+) of (E)-2-cyano-3-(3,4-dihydroxy-5-nitrophenyl)-N, N-di(prop-2-yn-1-yl) acrylamide (EB-11-38)

REFERENCES

1. Schindelin J, Arganda-Carreras I, Frise E, Kaynig V, Longair M, Pietzsch T, et al. Fiji: an open-source platform for biological-image analysis. *Nat Methods*. 2012;9(7): 676–82. doi: https://doi.org/10.1038/nmeth.2019

2. Deshpande PB, Randey, A. K., Dhameliya, D. R., Rathod, B. D., & Luthra, P. K, inventor; Alembic Ltd, assignee. Process for the preparation of entacapone (U.S. Patent Application No. US20100234632A1). U.S2010.

3. Dziuba D, Pohl R, Hocek M. Polymerase synthesis of DNA labelled with benzylidene cyanoacetamide-based fluorescent molecular rotors: fluorescent light-up probes for DNA-binding proteins. *Chem Commun (Camb)*. 2015;51(23): 4880–2. doi: https://doi.org/10.1039/c5cc00530b

4. Tran DN, Blaszkiewicz C, Menuel S, Roucoux A, Philippot K, Hapiot F, et al. Using click chemistry to access mono- and ditopic beta-cyclodextrin hosts substituted by chiral amino acids. *Carbohydr Res*. 2011;346(2): 210–8. doi: https://doi.org/10.1016/j.carres.2010.11.024
